# Multi-Antibiotic Porous Systems for Tailored Drug Delivery in Dentistry: Formulation Strategy, Physicochemical Properties, and Release

**DOI:** 10.3390/pharmaceutics18040409

**Published:** 2026-03-26

**Authors:** Monika Biernat, Anna Sylla, Krzysztof Adam Stępień, Joanna Giebułtowicz, Lidia Ciołek, Piotr Szterner, Paulina Tymowicz-Grzyb, Bartosz Kózka, Dorota Olczak-Kowalczyk

**Affiliations:** 1 Biomaterials Research Group, Lukasiewicz Research Network Institute of Ceramics and Building Materials, Cementowa 8, 31-983 Cracow, Poland; anna.sylla@icimb.lukasiewicz.gov.pl (A.S.); lidia.ciolek@icimb.lukasiewicz.gov.pl (L.C.); piotr.szterner@icimb.lukasiewicz.gov.pl (P.S.); paulina.tymowicz@icimb.lukasiewicz.gov.pl (P.T.-G.); bartosz.kozka@wum.edu.pl (B.K.); 2 Department of Drug Chemistry, Pharmaceutical and Biomedical Analysis, Medical University of Warsaw, 02-091 Warsaw, Poland; krzysztof.stepien@wum.edu.pl (K.A.S.); joanna.giebultowicz@wum.edu.pl (J.G.); 3 Department of Pediatric Dentistry, Medical University of Warsaw, 02-091 Warsaw, Poland; dorota.olczak-kowalczyk@wum.edu.pl

**Keywords:** multi-antibiotic system, porous composites, drug carriers, formulation strategy, antibiotic release

## Abstract

**Background/Objectives**: Although triple antibiotic paste is effective in managing infected primary teeth, its incomplete removability from tooth structure remains a major limitation, prompting the search for alternative drug-delivery systems. The aim of this study was to obtain a multi-antibiotic porous composite system for tailored drug delivery, to develop a formulation strategy, and to characterize the physicochemical properties and drug release. **Methods**: The developed composites consisted of a porous composite matrix (PCM; chitosan/bioactive filler) and two or three antibiotics (ciprofloxacin [CIP], metronidazole [MET], clindamycin [CLI]). Three methods of incorporating antibiotics were used: applying an antibiotic solution to the stabilized PCM; introducing an antibiotic solution into the polymer matrix; and introducing an antibiotic into the polymer matrix as nanoparticles. The physicochemical properties of the composites, including microstructure, compressive strength, and swelling, were assessed. The antibiotic release profile was assessed for up to 168 h. **Results**: The most advantageous method for introducing MET and CLI, in terms of release profile, was applying them to the PCM surface, whereas ciprofloxacin exhibited stable release when incorporated directly into the polymer matrix and entrapped during the stabilization process. The composites with nanoparticles, including MET or CIP, did not release any active substances during the experimental period. **Conclusions**: The results demonstrate that the developed formulation strategy enables the production of composites that rapidly release substantial amounts of the active substances within a short time frame and maintain their concentration for an extended period, which may be beneficial for the treatment of bacterial infections.

## 1. Introduction

Triple antibiotic paste (TAP), composed of metronidazole, ciprofloxacin, and minocycline, has been widely reported as an effective antimicrobial strategy for the management of infected primary teeth. Although the latest research focuses on the complete or partial exclusion of antibiotics, TAP treatment is still used. High clinical and radiographic success rates have been documented for its use in lesion sterilization and tissue repair (LSTR) [1,2]. In LSTR-based protocols, mechanical instrumentation of root canals is avoided, and the medicament is placed only on the pulpal chamber floor, significantly reducing treatment time and the number of clinical visits. This simplified approach is particularly advantageous in pediatric patients with limited cooperation and in settings with restricted clinical resources [3]. Since minocycline’s clinical use is associated with tooth discoloration and potential cytotoxic effects, modified formulations have been developed that replace minocycline with alternative antibiotics, such as clindamycin, while maintaining broad-spectrum antimicrobial activity [4].

Despite its clinical effectiveness, a major technical limitation of TAP is the difficulty of its complete removal from the tooth structure. Even intensive irrigation protocols using sodium hypochlorite, EDTA, or ethanol fail to eliminate the material entirely from dentinal irregularities. Passive ultrasonic irrigation with NaOCl improves removal efficiency; however, clinically relevant residues remain [5]. The persistence of TAP may adversely affect subsequent treatment outcomes, as residual material can interfere with the adhesion of definitive restorative materials or resin composites [6]. Moreover, elevated local concentrations of retained antibiotics may exert cytotoxic effects on apical papilla stem cells, potentially compromising tissue healing and regeneration [5]. Therefore, alternative drug-delivery systems for this application are under active investigation.

Researchers believe that hydrogels and hydrogel-based materials, which can absorb significant amounts of fluids and swell, play a special role in drug delivery. These materials can release active substances through diffusion or in response to specific environmental factors, e.g., pH, temperature changes, or enzyme activity [7]. Depending on the hydrogel carrier type and form, the active substance, and the formulation strategy, drug release may follow a burst or sustained release profile. Typically, loaded hydrogel carriers are obtained by directly delivering the active substance to pre-crosslinked hydrogels or by encapsulating the drug within the hydrogel matrix. By introducing an additional release system, such as micro- or nanocapsules, drug release can be delayed and slowed [7].

The use of polymeric nanocarrier systems is a key strategy for modulating the kinetics of antibiotic release. Thanks to their unique physicochemical properties, certain polymer-based nanomaterials may help mitigate specific bacterial resistance mechanisms or facilitate penetration through biofilm matrices; however, these effects are highly dependent on the material composition and surface characteristics of the nanoparticles, and cannot be generalized to all polymeric nanocarriers [8,9]. In the encapsulation of active substances in polymer nanoparticles (NPs), the primary goal is to achieve a stable, prolonged release profile [8]. Among available biomaterials, poly(lactic-co-glycolic acid) (PLGA) is the most widely used, primarily due to its full biocompatibility and FDA approval for medical use. In a biological environment, PLGA undergoes gradual hydrolytic degradation into lactic and glycolic acids—metabolites that are naturally eliminated by the body [9].

Among the many polymers capable of forming hydrogel drug carriers, chitosan is a particularly promising material [7,10,11,12]. It is a natural, biocompatible, non-toxic polysaccharide that does not elicit an immune response. It is readily available and inexpensive, produced by deacetylating chitin from sources such as crab and shrimp shells. Chemically, it is composed of randomly distributed β-(1-4)-conjugated units of D-glucosamine and N-acetyl-D-glucosamine. The presence of amine and hydroxyl functional groups in its molecular structure confers reactivity, cross-linking capacity, and the potential for numerous modifications. Furthermore, chitosan also possesses antioxidant and antimicrobial properties, is biodegradable, susceptible to enzymatic degradation, and can be sterilized, making it an excellent material for biotechnological applications [13,14].

Many researchers suggest that, due to chitosan’s unique properties, carriers of active substances based on this polymer show promising results in dentistry in various forms [15,16,17,18]. Porous chitosan structures in sponge form may be particularly important for dental applications. Due to the cationic character of chitosan, enabling the absorption and a large internal pore surface area, these materials can absorb significant amounts of fluids, allowing for the entrapment and release of a wide range of active substances [19,20]. Given the broad control over crosslinking density and pore size, and the potential to modify and enrich chitosan materials with various additional particles (e.g., ceramics or bioglasses), chitosan porous composites appear to be valuable carriers for precisely tuning release kinetics in dentistry.

The aim of the work was to develop and obtain a multi-antibiotic porous composite system for tailored drug delivery in dentistry. Chitosan and a ceramic filler, a mixture of bioglass and BaSO_4_, were used to synthesize a porous composite matrix (PCM) that serves as the basis for the antibiotic carrier. The authors have previously used the porous chitosan/bioglass system as an effective peptide carrier for orthopedic applications [21]. A key element of such a system is the innovative bioglass with a specifically selected composition and grain size, capable of releasing zinc ions with bactericidal activity and supporting therapeutic effects over a longer period. The innovation in the PCM system lies in the addition of BaSO_4_ as a radiopacity agent, which is significant for future dental applications.

The objective of the work was to develop a drug-carrier formulation strategy that could ensure the synergistic effect of various antibiotics in the intended use of the resulting composite carrier. Considering that an effective drug carrier should have an appropriate release profile, ensuring local, immediate, and sustained release of these active substances, three types of composites containing two or three antibiotics, introduced via various methods and forms, were designed for comparison.

The study used three antibiotics recommended by the American Academy of Pediatric Dentistry as components of a three-antibiotic paste for LSTR: ciprofloxacin, metronidazole, and clindamycin.

Taking into account the advantages of using nanoparticles as drug carriers, the aim of this work was also to develop, obtain, and characterize nanoparticles containing two of the three selected antibiotics, incorporate them into the hydrogel composite system, and determine their effect on the physico-mechanical properties and drug release.

The innovation of this work was the introduction of three different antibiotics, in different ways, into a porous composite matrix based on chitosan and bioglass, and the study of the physicochemical properties of these systems, as well as the antibiotic release profile from them. At least two distinct methods for incorporating each selected antibiotic into the composite formulation were developed. To the best of our knowledge, no such solution has been described in the literature, making the results valuable for further consideration and application.

## 2. Materials and Methods

### 2.1. Materials

The composites were obtained from: Chitoceutical chitosan 95/2000 (degree of deacetylation ≥92.6%) (HMC+—Heppe Medical Chitosan GmbH company, Halle, Germany), tetraethoxysilane, zinc nitrate(V) hexahydrate, calcium nitrate(V) tetrahydrate (Avantor Performance Materials Poland S.A., Gliwice, Poland), triethyl phosphate(V) (Fluka, Chemie GmbH, Buchs, Switzerland) and barium sulfate (Chempur, Piekary Slaskie, Poland), 99.8% acetic acid (Merck KGaA, Darmstadt, Germany), HPLC-grade acetonitrile (Merck KGaA, Darmstadt, Germany), HPLC-grade formic acid (Merck KGaA, Darmstadt, Germany), sodium hydroxide (Chempur, Piekary Slaskie, Poland), antibiotics: ciprofloxacin (CIP) (Mw = 331.34 Da, TRC-C482500, LGC Standards Sp. z o. o., Kielpin, Poland), metronidazole (MET) (Mw = 171.16 Da, TRC-M338880, LGC Standards Sp. z o. o., Kielpin, Poland), clindamycin (CLI) (Mw = 424.98 Da, CAS: 18323-44-9; Targetmol Chemicals Inc., Wellesley Hills, MA, USA).

Nanoparticles with an antibiotic were obtained from: poly(vinyl alcohol) (PVA): Mw 13–23 kDa, 98% hydrolyzed (Merck KGaA, Darmstadt, Germany), RESOMER RG 502 poly(D, L-lactide-co-Glycolide) (PLGA): specific viscosity of 0.16–0.24 (dL/g), a lactide to glycolide ratio of 50:50, and a terminal ester group (Evonik Operations GmbH, Essen, Germany), dichloromethane analaR NORMAPUR Reag. Ph.Eur., ACS (VWR International S.A.S. Rosny-sous-Bois, France), 88% lactic acid p.a. (POCH, Gliwice, Poland), 96% *v/v* ethanol p.a. (Chempur, Piekary Slaskie, Poland).

Materials used for research: Saline solution—NaCl 0.9% (B. Braun Melsungen AG, Melsungen, Germany), ciprofloxacin-d8 (Mw = 339.39 Da, TRC-C482501, LGC Standards Sp. z o. o., Kielpin, Poland), metronidazole-d3 (Mw = 174.17 Da, TRC-M978800-10MG, LGC Standards Sp. z o. o., Kielpin, Poland), clindamycin-13C, D3 (Mw = 428.99 Da, TRC-C579992-1MG, LGC Standards Sp. z o. o., Kielpin, Poland). Ultrapure water was obtained from a Millipore water purification system (Milli-Q water, Darmstadt, Germany).

All other materials used in the syntheses and in the research methods are described in the preparative sections.

### 2.2. Preparation of Bioactive Filler

Bioglass was synthesized via the sol–gel method. The glass composition was 70 wt% SiO_2_, 23 wt% CaO, 5 wt% P_2_O_5_, and 2 wt% ZnO; its properties were described in a previous publication [22]. During synthesis, the precursor mixture was heated in an incubator, with the temperature increased from 40 °C to 180 °C. The dried gel was heated in an electric furnace at 650 °C. The dried gel was then ground to a particle size of 90% below 86.972 μm. The ground bioglass was placed in an agate mortar and mixed with a radiopacity agent (BaSO_4_).

### 2.3. Preparation of Nanoparticles with Antibiotics

Ciprofloxacin nanoparticles (npCIP): First, the aqueous phase was prepared. For this purpose, 200 mg of ciprofloxacin was weighed and dissolved in 10 mL of 1% lactic acid. Next, the oil phase was prepared by dissolving 1000 mg of PLGA in 10 mL of dichloromethane (DCM). The first aqueous phase was added to the oil phase, and the mixture was emulsified using a sonication probe (Vibra-Cell VCX 750, Sonics, Newtown, CT, USA) at 38% amplitude in an ice bath for 32 s (pulse on 4 s, pulse off 7 s), producing a *w*/*o* emulsion. Next, the second aqueous phase was prepared by dissolving 1 g of PVA in 200 mL of deionized water. The *w*/*o* emulsion was then added to the PVA solution and immediately sonicated for 63 s (pulse on for 4 s, pulse off for 7 s) at an amplitude of 38% in an ice bath to prepare a *w*/*o*/*w* emulsion. The organic phase from *w*/*o*/*w* was evaporated using a magnetic stirrer at 250 rpm at room temperature for 22.5 h. The recovered nanoparticles were washed three times with water (200 mL each time) to remove PVA (3 × 30 min at 4700 rpm at 18 °C). After each washing step, the supernatant was visually inspected. Only transparent, non-turbid supernatants—indicating the absence of dispersed material—were discarded. Finally, 15 mL of deionized H_2_O was added, and the mixture was sonicated for 10 min. An additional 10 mL of deionized water was added, followed by vortexing for 30 s (20 × 100 rpm). The sample was then transferred to an open vessel and freeze-dried using a Beta model 1–16 (Martin Christ GmbH, Osterode am Harz, Germany).

Metronidazole nanoparticles (npMET): First, the aqueous phase was prepared. For this purpose, 0.42 g of PVA was dissolved in 140 mL of deionized water. Then, 342.2 mg of metronidazole was dissolved in a mixture of 11.42 mL of dichloromethane (DCM) and 2.58 mL of 96% ethanol (EtOH). The mixture was stirred on a magnetic stirrer until completely mixed. Next, 1.2 g of PLGA was dissolved in the antibiotic solution under magnetic stirring (oil phase). The oil phase was then added to the aqueous phase and emulsified using a sonication probe at 38% amplitude in an ice bath for 90 s (pulse on for 4 s, pulse off for 7 s). The organic phase was evaporated using a magnetic stirrer at 250 rpm at room temperature for 21 h. The recovered nanoparticles were washed three times with water (200 mL each time) to remove PVA (3 × 30 min at 4700 rpm at 18 °C). After each washing step, the supernatant was visually inspected. Only transparent, non-turbid supernatants—indicating the absence of dispersed material—were discarded. Finally, 15 mL of deionized H_2_O water was added, and the mixture was placed in an ultrasonic bath for 10 min. An additional 10 mL of deionized water was added, then the mixture was centrifuged for 30 s (20 × 100 rpm). The sample was then transferred to an open vessel and freeze-dried.

### 2.4. Physicochemical Characterization of Nanoparticles

Structures of the obtained nanoparticles were characterized using scanning electron microscopy (SEM). Imaging was performed under high vacuum conditions using a TLD detector at an accelerating voltage of 100 kV. Before imaging, the sample was coated with a thin conductive layer (10 nm of gold).

The Zeta potential, polydispersity index (PDI), and particle size distribution of nanoparticles were measured using a Zetasizer Nano ZS (Malvern Instruments, Malvern, UK) with dynamic light scattering (DLS). The measurements were performed on suspensions at 0.01 mg·mL^−1^ in deionized water or 1% CH_3_COOH at 25 °C. The refractive indices of the dispersing medium and PLGA were set at 1.33 and 1.46, respectively. Each value was obtained as the average of three consecutive measurements performed with the device, each including at least three measurements and a minimum of 10 runs. The zeta potential was calculated based on the Smoluchowski equation for electrophoretic mobility.

UV–Vis spectroscopy was used to quantitatively determine CIP and MET during drug loading and encapsulation/release efficiency tests. Four calibration curves were prepared. For drug loading and encapsulation efficiency, CIP was completely dissolved in 1% CH_3_COOH, and MET was completely dissolved in deionized water. The calibration curve for the release studies was obtained by completely dissolving CIP or MET in 0.9% NaCl (B. Braun Melsungen AG, Melsungen, Germany). For all curves (regardless of the medium), different concentrations ranging from 50 to 0.78 μg/mL (CIP) and from 250 to 3.9 μg/mL (MET) were prepared from stock solutions (those with the highest indicated concentration). λ_max_ was identified at 279 nm (for CIP in 1% CH_3_COOH), 274 nm (for CIP in NaCl), and 321 nm (for both MET). The absorbance of each dilution was measured using λ_max_ to plot a calibration curve.

Drug loading (DL) [%] and encapsulation efficiency (EE) [%] were measured according to the protocol described in the literature [23]. 10 mg of nanoparticles were dissolved in 10 mL of dichloromethane (3 replicates were prepared). Then, 10 mL of 1% w/w acetic acid (CIP) or deionized water (MET) was added, and the mixture was stirred for 6 h on a magnetic stirrer. After this time, 200 µL of the aqueous phase was collected to measure the antibiotic concentration (four replicates per sample). The absorbance of the aqueous phase was measured at 279 nm (for CIP) or 321 nm (for MET) using a 96-well quartz plate with a microplate reader equipped with a UV/VIS spectrometer (VANTAstar™, BMG LABTECH, Ortenberg, Germany). The drug loading degree and encapsulation efficiency were calculated using the following equations:

(1) $$DL\left[\%\right]=\frac{drug\;weight\;in\;nanoparticles}{total\;weight\;of\;nanoparticles}\times 100$$

(2) $$EE\left[\%\right]=\frac{drug\;remained\;in\;the\;nanoparticles}{feeding\;weight\;of\;drug}\times 100$$

The kinetics of antibiotic release from nanoparticles were determined by placing 12 mg of particles in the upper part of Costar^®^ Spin-X^®^ Centrifuge Tube Filters (with a 0.22 µm pore CA membrane) (Corning, Inc., New York, NY, USA). Three replicates were prepared for each sample. The lower part of the filter was filled with 1.1 mL of 0.9% NaCl solution (B. Braun Melsungen AG, Melsungen, Germany), and 0.7 mL of 0.9% NaCl solution was placed in the upper part (total volume of NaCl was 1.8 mL per sample). The pH of the solution before any tests was measured in three independent replicates. The samples were then placed in an incubator with gentle shaking (37.5 °C, 75 rpm). Measurements were taken after 1 h, 3 h, and on days 1, 2, 4, 7, and weekly until day 49. After this time, 300 µL of solution (from the lower part of the filter) was collected to measure the antibiotic concentration (measurements were performed twice for each sample—300 µL was collected twice). Then, fresh saline (600 µL) was added to the lower part. The immersion conditions were maintained throughout the experiment. The absorbance of the solution was measured at 274 nm (for CIP) or 321 nm (for MET) using a 96-well quartz plate with a microplate reader with a UV/VIS spectrometer (VANTAstar™, BMG LABTECH).

### 2.5. Preparation of Multi-Antibiotic Porous Composites

**Figure 1:**
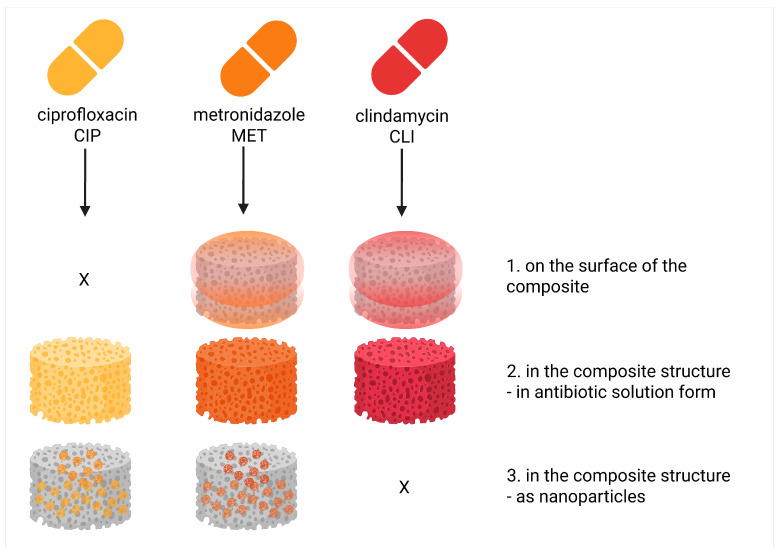
Strategy for incorporating antibiotics into a chitosan porous composite matrix.

In this study, three types of antibiotic-containing composites were obtained. All composites consisted of a porous composite matrix (PCM; chitosan/bioactive filler) and a system of two or three antibiotics introduced in different ways. The porous structure of the composites was obtained by lyophilizing stable filler dispersions in chitosan solutions. The process was carried out in a Christ Epsilon 2-16D shelf freeze-dryer. After freezing the dispersion to −35 °C, the pressure was reduced to 40 Pa, and the main drying stage was carried out, during which the solvent sublimated. During the post-drying stage, the pressure in the freeze-dryer chamber was reduced to 25 Pa. The dispersion composition was selected to yield a chitosan/filler weight ratio of 1/1, with a polymer concentration of 2% by weight. Depending on the type of antibiotic and the strategy of its incorporation into the composite matrix (Figure 1), the procedures differed in some production conditions, dispersion components, and the order of components’ addition. All porous composites obtained by lyophilization were stabilized in ethanol (96%), neutralized in 0.1 M NaOH, and freeze-dried again.

All composites were sterilized by fast-electron-beam irradiation at the Institute of Chemistry and Nuclear Technology in Warsaw, Poland. The irradiation parameters were set as follows: a dose of 28 kGy, a conveyor speed of 0.462 m/min, and a beam current of 600 mA.

Procedure 1—composites with antibiotic(s) applied to the surface of the porous structure. The filler dispersion in a 1% acetic acid solution of chitosan, prepared by magnetic stirring (15 min, 160 rpm), was lyophilized to obtain PCM. After stabilization and drying, antibiotic(s) were applied to the porous structure of PCM. For this purpose, a solution of the selected antibiotic(s) (metronidazole and/or clindamycin) at the appropriate concentration was prepared in a 50:50 (*v*/*v*) mixture of ethanol and deionized water. The antibiotic solution was then applied to the PCM surface, and after 18 h of refrigerated incubation, the entire structure was freeze-dried again. The amount of antibiotic(s) applied as a solution per 1 g of porous composite, and the concentrations of the antibiotic solutions used for each composite are presented in Table 1.

Procedure 2—composites with antibiotic (antibiotic solution form) introduced in situ into the composite matrix, and antibiotic(s) applied to the surface of the porous structure. A dispersion created by magnetic stirring of the filler, chitosan solution, and antibiotic was lyophilized. The dispersion preparation process varied by antibiotic type. For clindamycin, a stable dispersion of the bioactive filler was first obtained in a chitosan solution with 1% acetic acid. Then, the clindamycin solution in 1% acetic acid was added, and the mixture was stirred magnetically for 10 min (100 rpm). For ciprofloxacin or metronidazole, the antibiotic was first dissolved in 1% acetic acid, and the chitosan was then dissolved in the prepared antibiotic solution for 18 h at 5 °C. After that, the filler was added, and the dispersion was prepared by magnetic stirring (15 min, 100 rpm). The amounts of antibiotic introduced into the composite matrix per 1 g of individual porous composite, along with the concentrations of the antibiotic solutions used, are presented in Table 1. The subsequent procedure for obtaining the composites is identical to Procedure 1, including the application of additional antibiotic(s) to the surface of the porous structure. The amount of antibiotic(s) applied in solution per 1 g of porous composite and the concentrations of the antibiotic solutions used for each composite are presented in Table 1.

Procedure 3—composites with an antibiotic introduced into the composite matrix in antibiotic solution form, an antibiotic introduced into the composite matrix in the form of nanoparticles, and antibiotic(s) applied to the surface of the porous structure. Dispersions created by magnetic mixing of the filler, chitosan solution, and antibiotic (antibiotic solution form and antibiotic nanoparticles) were lyophilized. The antibiotic (in its free base form) was dissolved in 1% acetic acid, and the nanoparticles were then added to the resulting solution. These were dispersed by sonication (disintegration using a sonication probe at 65% amplitude in an ice bath for 32 s; pulse on 4 s, pulse off 7 s). Chitosan was then dissolved in the obtained solution of the antibiotic, along with the antibiotic polymeric nanoparticles, for 18 h at 5 °C. In the next step, the filler was added, and the dispersion was obtained via magnetic mixing (15 min, 100 rpm). The amounts of antibiotics introduced into the composite matrix, in nanoparticle and solution forms, per 1 g of porous composite, and the concentrations of the antibiotic solutions used are presented in Table 1. The further procedure for obtaining composites is identical to procedures 1 and 2. The amount of antibiotic applied per 1 g of porous composite, as a solution, and the concentrations of the antibiotic solutions used for individual composites are presented in Table 1.

### 2.6. Physicochemical Characterization of Porous Composites

#### 2.6.1. Porous Microstructure Characterization (SEM)

Imaging of composites was performed using a field-emission scanning electron microscope (Nova NanoSEM 200, FEI, Lausanne, Switzerland). Microstructure was characterized under high-vacuum conditions using an ETD detector at an accelerating voltage of 10 kV. The samples were covered with conductive material (10 nm gold film) before the imaging, using a sputter coater (Leica EM SCD500, Wetzlar, Germany). The minimum, maximum, and mean pore size were determined, as well as the pore size distribution, via calculation from 170 pore measurements from SEM images.

#### 2.6.2. Mechanical Compressive Strength

The mechanical properties of the porous composites were assessed using compression tests. All samples were prepared as cylindrical samples approximately 7–9 mm in height and 12–15 mm in diameter. Compression tests were performed on four samples from each series at a traverse speed of 1 mm/min on a Zwick Roell 5 kN ProLine testing machine (Ulm, Germany), with strain levels of 1%, 5%, 10%, 20%, and 50% (maximum). Measurements were performed for dry and wet samples soaked in physiological saline. The Strength retention after wetting was calculated using the following equations:

(3) $$Strength\;retention\left[\%\right]=\frac{Compressive\;Stress\;in\;wet\;condition}{Compressive\;Stress\;in\;dry\;condition}\times 100$$

#### 2.6.3. Swelling Test

The swelling capacity was evaluated in seven periods of time: 1 h, 4 h, 24 h (1 day), 168 h (7 days), 336 h (14), 504 h (21 days), 672 h (28 days) by determination changes in weight the samples before (w) and after incubating (w_s_) in the 0.9% NaCl with pH = 6.99. The swelling capacity was determined as the value [%] of the ratio of weight increase (w_s-w_) relative to initial weight (w). Each value was calculated as the mean of 5 independent measurements.

### 2.7. Antibiotics Releasing from Composites

Release studies were carried out using a VK 7025 dissolution apparatus (Varian; Erweka GmbH, Heusenstamm, Germany) in compliance with the requirements of the Polish Pharmacopeia XIII. A 0.9% (*w*/*v*) aqueous sodium chloride solution (300 mL) was used as the release medium. The experiments were carried out at 37 ± 0.5 °C under limited light exposure for 7 days. Samples of the release medium were collected at 0.25, 0.5, 1, 2, 4, 6, 12, 24, 48, 72, 96, 144, and 168 h. The withdrawn volume was replaced with fresh medium to maintain sink conditions. Quantitative analysis was performed using an Agilent 1260 Infinity LC system (Agilent Technologies, Santa Clara, CA, USA) coupled with a QTRAP 4000 mass spectrometer (AB Sciex, Framingham, MA, USA). Chromatographic separation was achieved on a BionaCore C18 UFPLC column (Bionacom Limited, Coventry, UK) (4.6 mm × 100 mm, 2.7 µm) maintained at 40 °C, with the autosampler set to 4 °C and an injection volume of 10 µL. The mobile phases consisted of (A) water with formic acid (998:2, *v*/*v*) and (B) acetonitrile with formic acid (998:2, *v*/*v*), delivered at a flow rate of 750 µL min^−1^. Gradient elution was applied as follows: 0.00–1.00 min, 95% A/5% B; 5.00–9.00 min, 5% A/95% B; 9.10–12.00 min, 95% A/5% B. Mass spectrometric detection was carried out using an electrospray ionization (ESI) source operated in the positive ion mode with the following parameters: source temperature 600 °C, capillary voltage 5000 V, curtain gas 35 psi, collision gas set to medium, GS1 60 psi, and GS2 40 psi. Quantification was performed in the multiple reaction monitoring (MRM) mode using the following Q1 → Q3 transitions: metronidazole 172 → 82, ciprofloxacin 332→90, clindamycin 425 → 377, and the corresponding deuterated internal standards metronidazole-D4 176 → 82, ciprofloxacin-D8 340 → 296, and clindamycin-D3 428→85. The optimized MRM parameters (DP/CE/CXP) were 41 V/10 V/37 V for metronidazole, 81 V/10 V/33 V for ciprofloxacin, 91 V/10 V/29 V for clindamycin, 61 V/37 V/6 V for metronidazole-D4, 91 V/29 V/16 V for ciprofloxacin-D8, and 96 V/123 V/6 V for clindamycin-D3.

### 2.8. Statistical Analysis

Statistical analysis for microstructure characterization, mechanical compressive test, and swelling test was performed using STATISTICA 13 software (license: Medical University of Warsaw). Differences between groups were analyzed using ANOVA with Tukey’s post hoc test for equinomureos groups, or the Kruskal–Wallis test for unequal groups. The assumptions of ANOVA (normality and homogeneity of variances) were verified using the Shapiro–Wilk test and Bartlett’s test, respectively. The significance level (*p*-value) for the above analyses was set at 0.05.

For antibiotic-releasing tests, the difference factor (f_1_) and similarity factor (f_2_) were used to compare the drug dissolution or release profiles obtained for different formulations. The f_1_ factor expresses the percent difference between two release profiles at each sampling time point. Values off_1_ between 0 and 15 indicate that there is no significant difference between the compared profiles. The f_2_ factor is a logarithmic measure used to evaluate the similarity between two dissolution profiles; f_2_ values in the range of 50–100 indicate similar drug-release behavior.

## 3. Results

### 3.1. Synthesis and Characterization of Nanoparticles with Antibiotics

PLGA nanoparticles (nps) containing ciprofloxacin (npCIP) or metronidazole (npMET) were successfully obtained using two solvent evaporation methods: *w*/*o*/*w* and *o*/*w*. Their physicochemical properties, including particle sizes determined by SEM, particle size distribution, zeta potential, polydispersity index (PDI) determined by DLS, drug loading, and encapsulation efficiency, are shown in Table 2. The zeta potential (ζ) indicated good electrostatic stability greater than ±30 mV. The zeta potential of synthesized nps in water was −34.8 ± 1.7 mV for particles with ciprofloxacin and −38.5 ± 2.9 for particles with metronidazole. The zeta potential of synthesized nps in 1% CH_3_COOH was −1.7 ± 0.5 mV for particles with ciprofloxacin and −3.3 ± 0.2 mV for particles with metronidazole. The determined drug loading for nanoparticles with ciprofloxacin is 0.10 ± 0.02%, and the determined encapsulation efficiency is 0.60 ± 0.10%. The determined drug loading for nanoparticles with metronidazole is 0.76 ± 0.10%, and the determined encapsulation efficiency is 3.41 ± 0.46%.

**Figure 2:**
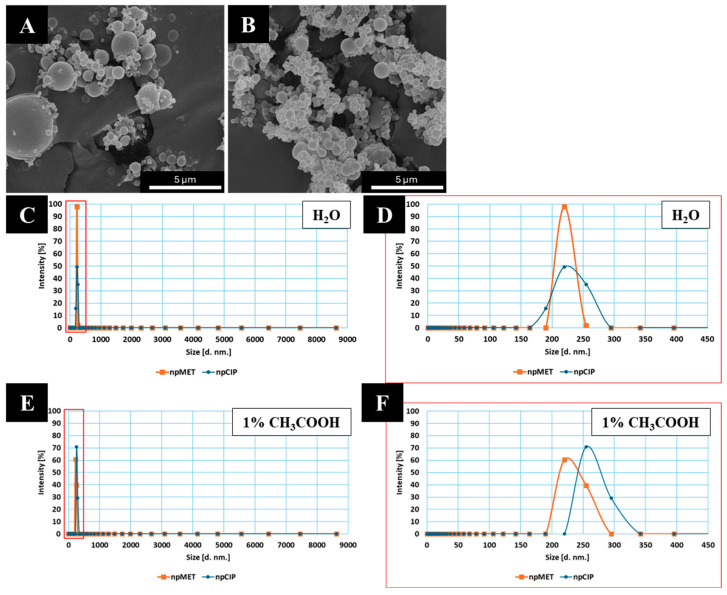
SEM images of PLGA nanoparticles with (**A**) ciprofloxacin and (**B**) metronidazole at 20,000× magnification. (**C**) Nanoparticle size distribution based on DLS measurements in water of nanoparticles with ciprofloxacin or metronidazole. (**D**) A zoomed view of (**C**), showing the nanoparticles’ size from 0 to 450 d. nm. (**E**) Nanoparticle size distribution based on DLS measurements in acetic acid of nanoparticles with ciprofloxacin or metronidazole. (**F**) A zoomed view of (**E**), showing the nanoparticles’ size from 0 to 450 d. nm.

SEM images (Figure 2) of the prepared nanoparticles containing: (A) ciprofloxacin or (B) metronidazole reveal a spherical morphology with a smooth surface. Based on the results obtained from the particle size distribution analyzer (analysis in water), the particle size of npCIP (Figure 2C,D, Table 2) is distributed from 199.6 ± 41.5 nm (D0.1) to 305.2 ± 70.0 nm (D0.9) with a median diameter of 246.9 ± 53.7 nm (D0.5). While the average Z-size (Z-Ave) is 1279.8 ± 409.2 nm, and PDI is 0.85 ± 0.14. The particle size distribution in acetic acid (Figure 2E,F, Table 2) is similar to that obtained for deionised H_2_O. However, Z-ave is almost 1.7 times smaller, and the PDI is 0.66 ± 0.16. The size distribution of npMET (Figure 2C,D, Table 2) in deionised water ranges from 169.1 ± 64.8 nm (D0.1) to 328.8 ± 83.3 nm (D0.9) with a median diameter of 262.0 ± 53.2 nm (D0.5). The average Z-size is 1743.4 ± 268.1 nm, with a PDI of 0.96 ± 0.07. The particle size distribution in acetic acid (Figure 2E,F, Table 2) is also similar to that obtained for deionised H_2_O. However, Z-ave is almost 3.0 times smaller, and the PDI is 0.66 ± 0.11. The PDI values reveal a highly polydisperse distribution type in all cases. However, they are lower when measured in acetic acid (Table 2).

**Figure 3:**
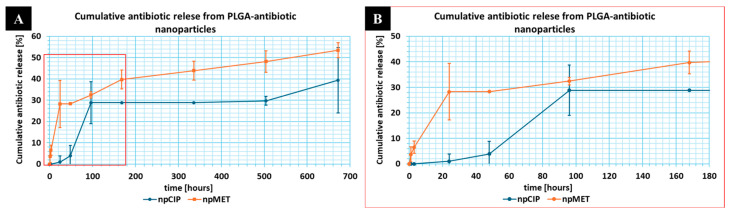
(**A**) Cumulative curves of antibiotics release from nanoparticles; (**B**) a zoomed view of (**A**), showing the period from 0 to 168 h.

The kinetics of antibiotic release from the nps were evaluated in a saline environment (0.9% NaCl, pH 6.99). Figure 3 illustrates the cumulative antibiotic release (%) over time. The npCIP formulation exhibited a complex, biphasic release profile. An initial burst release, during which approximately 29% of the loaded drug was released over the first 96 h (4 days) (Figure 3B), was followed by a sustained phase lasting up to 672 h (28 days). Metronidazole was characterized by a more rapid, biphasic release in significantly higher quantities (Table A1). A burst release can be observed, with approximately 28% of the loaded drug released in 24 h, followed by sustained release, with cumulative antibiotic release at approximately 53% after 28 days. Notably, both systems demonstrated the capacity for prolonged, gradual release throughout the entire 28-day observation window.

### 3.2. Synthesis and Characterization of Chitosan Composites with Antibiotics

#### 3.2.1. Strategy of Incorporation of Antibiotics into Chitosan Composites

Composites with antibiotics based on porous composite matrix (PCM), consisting of chitosan, bioglass, and BaSO_4_, and enriched with three antibiotics: ciprofloxacin, metronidazole, and clindamycin, were successfully obtained.

**Figure 4:**
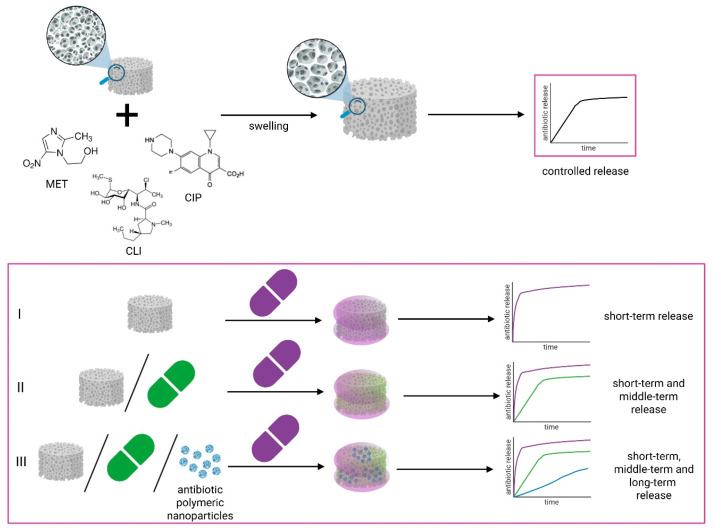
Design of the obtained drug-loaded porous chitosan composites and the idea of release.

The assumption was that the composites differed in the type of antibiotics and their incorporation ways, and thus their release rate (Figure 4):

(1) Composites in which antibiotics were applied to the surface of a porous PCM structure—with potential immediate but short-term release, as loading with antibiotics involves permeation of the previously stabilized PCM through the antibiotic solution.
(2) Composites in which antibiotics were applied to the PCM surface and additionally introduced in antibiotic solution form into the composite matrix, which combine short-term and middle-term release profiles, ensuring immediate and slightly extended release, as the incorporation of the antibiotic into the polymer matrix causes its entrapment and slows down its release.
(3) Composites in which antibiotics were applied to the PCM surface and additionally introduced into the composite matrix in two forms: antibiotic solution form and as antibiotic-containing polymer nanoparticles, which combine short-term, middle-term, and long-term release profiles with the potential for continuous antibiotic release from the initial administration to long periods associated with the release of the active substance from the nanoparticles.

The method of introducing a given antibiotic into the composite was largely influenced by the antibiotic’s solubility in water and other solvents, which introduced certain processing limitations.

Ciprofloxacin (free-base) is practically insoluble in water and polar organic solvents (up to 0.107 mg·mL^−1^ in acetone) [24]. In contrast, in acidic conditions, it is well soluble, e.g., in 0.1 N HCl at 25 mg·mL^−1^. Hence, we decided to introduce ciprofloxacin to the composite in two ways: (1) directly into the polymer matrix during its synthesis, where it will be introduced in antibiotic solution form as a solution in 1% acetic acid; (2) directly into the polymer matrix during its synthesis, incorporated in the form of polymeric nanoparticles synthesized based on PLGA.

Solubility of metronidazole in water is higher (12.3 mg·mL^−1^) [25], in ethanol slightly less (approx. 5 mg·mL^−1^). Hence, these properties allowed us to introduce metronidazole in three ways: (1) and (2) as above, and (3) directly onto the surface of porous polymer composite form as the solution in a water/ethanol *v/v* 1:1 mixture.

The solubility of clindamycin in water is not sufficient; it is enhanced by the addition of ethanol and some other hydrophilic solvents [26,27,28]. This feature did not allow us to administer the antibiotic as PLGA-based nanoparticles. The methods of introducing the selected antibiotics into the composites are presented in Figure 1. The types of composites obtained in this work, together with the method of introducing individual antibiotics and their content in the composites, are presented in Table 1.

#### 3.2.2. Porous Microstructure Characterization (SEM)

**Figure 5:**
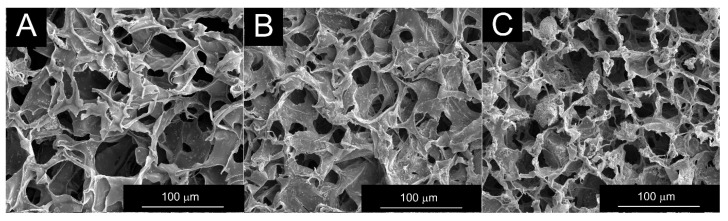
SEM images of three different types of composites with antibiotics: (**A**) composite PCM-MET50-CLI50 with antibiotics applied on the surface; (**B**) composite PCM/CIP0250-MET50-CLI50 with antibiotics applied on the surface and introduced into the composite matrix in the antibiotic solution form; (**C**) composite PCM/CIP0125/npCIP0125-CLI25 with antibiotics applied on the surface and introduced into the composite matrix in the antibiotic solution form and in the form of polymer nanoparticles.

The surface morphology of the obtained composites was examined using a scanning electron microscope. High-magnification (1000×) SEM images reveal some characteristic structural features of the three composite types (Figure 5). The filler grains (bioglass and BaSO_4_) present in the PCM, located in the walls of the pores, are clearly visible in all three composite types. In the composite with antibiotics applied on the PCM surface and antibiotics introduced as a solution into the polymer matrix, a slightly higher concentration of smaller grains can be observed in the pore walls than in the composite with antibiotics applied only on the surface. In the composite, in which antibiotics are introduced into the polymer matrix as nanoparticles, areas with the presence of polymer nanoparticles are visible.

**Figure 6:**
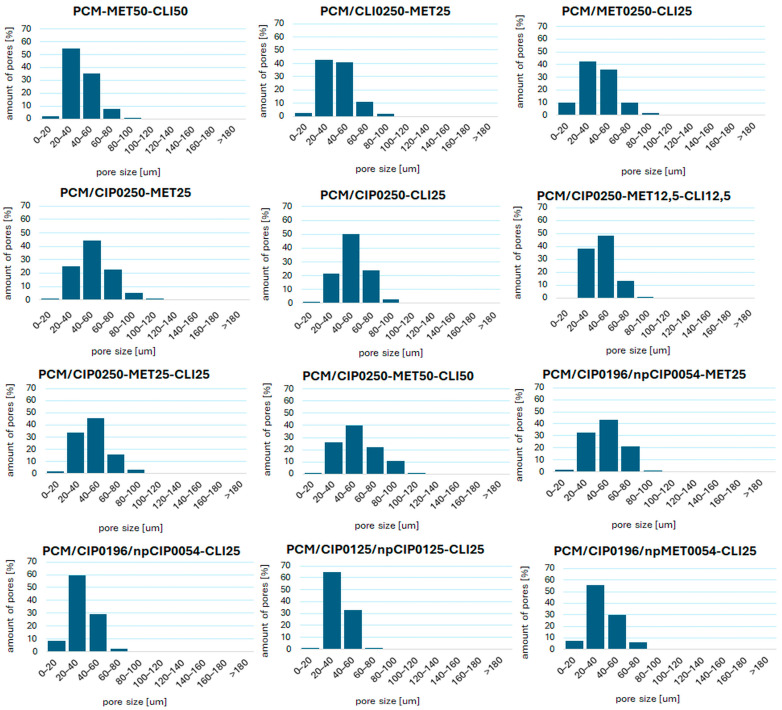
Pore size distribution of the obtained composites. Sample size for calculations: 170 pore measurements for each composite.

**Figure A1:**
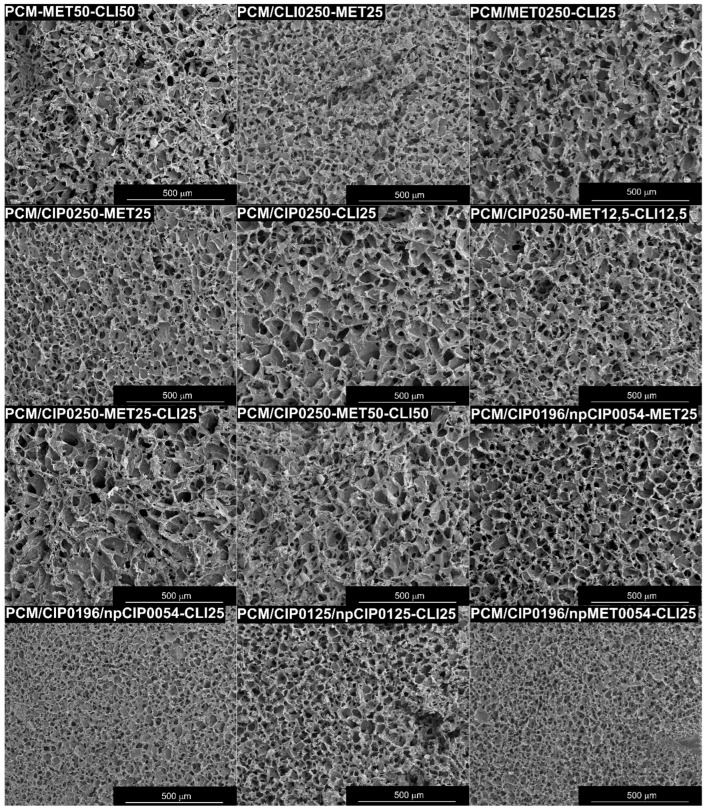
SEM images of the porous structure of the obtained composites.

SEM observations were also conducted at various magnifications to characterize the obtained composites in terms of porosity and pore size. All obtained composites exhibit open porosity throughout the sample volume (Figure A1), and their average pore size ranges from 35.53 ± 11.34 µm to 53.83 ± 18.65 µm (Table A2). The determined pore size distributions for all the composites considered are presented in Figure 6. All composites exhibit a regular pore-size distribution, with the largest proportion (65.88–97.65%) of pores in the 20–60 µm size range. It was observed that most of the obtained composites with antibiotics introduced simultaneously to the surface and into the polymer mass in antibiotic solution form have a predominant number of pores in the 40–60 µm range. The composite containing antibiotics on the surface, and most composites with antibiotics introduced as nanoparticles, have a predominant number of pores in the 20–40 µm range.

Comparing the five groups of composites specified in Table 1, they varied in terms of pore size from each other in some cases. Two groups of composites without nanoparticles, but with the antibiotic introduced into the composite matrix and with one or two antibiotics applied onto the porous composite surface, were statistically similar (*p* = 0.29). A likely reason for this similarity may be the effect of the antibiotic(s) deposited on the surface of the porous structure on pore size. The similarity was also observed for two groups of composites containing nanoparticles (*p* = 0.18), proving that the addition of nanoparticles has a similar effect on the microstructure of different porous composites. Additionally, similarity was detected between two groups of composites with nanoparticles and a composite with surface-applied antibiotics (*p* >> 0.05), which explains the observed similarity in pore size distribution across these groups.

Considering the similarities among individual composites within groups containing more than one composite, in the group of composites with two antibiotics introduced (on the surface and into the composite matrix in antibiotic solution form), no difference was detected between composites PCM/CLI0250-MET25 and PCM/MET0250-CLI25, and between composites PCM/CIP0250-MET25 and PCM/CIP0250-CLI25 (*p* = 0.23 and 0.75, respectively). Among the group of composites with three antibiotics introduced (two antibiotics on the surface and one into the composite matrix), no difference between composites PCM/CIP0250-MET12,5-CLI12,5 and PCM/CIP0250-MET25-CLI25 was observed (*p* = 0.58). In the group of composites with antibiotics including nanoparticles, the composites PCM/CIP0196/npCIP0054-CLI25 and PCM/CIP0125/npCIP0125-CLI25 were homogeneous (*p* = 0.39).

#### 3.2.3. Mechanical Compressive Strength

Compression tests were performed at strains of 1%, 5%, 10%, 20%, and 50% (maximum). Measurements were performed for dry and wet samples soaked in physiological saline. The compressive strength, both dry and wet, increases linearly with the deformation value (1, 5, 10, 20, 50%), reaching the highest value at max (50%).

Table 3 presents compressive stress (10% strain) values in dry and wet conditions for the obtained composites loaded with antibiotics (No. 1–12 in Table 3), and for blank carrier—PCM (porous composite matrix not loaded with antibiotic) (No. 0 in Table 3).

Compressive stress (10% strain) values in dry conditions were obtained in the range of 0.0642–0.088 MPa. Generally, these values were similar within the individual composite groups. However, in the case of groups of composites with two or three antibiotics (surface-applied and incorporated into the PCM as an antibiotic solution), some statistically significant differences occurred within a given group (Figure A3a). The highest compressive strength of all composites under dry conditions was recorded for the PCM/CIP0125/npCIP0125-CLI25 composite (0.088 MPa).

**Figure 7:**
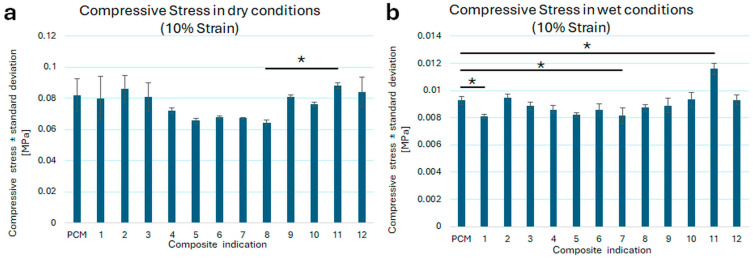
The values of compressive stress in dry and wet conditions (at 10% strain) for the obtained composites. Data represent four independent units of each composite (*n* = 4). Statistical analysis: (**a**) shows all statistically significant differences between the compressive strength values for all composites in dry conditions; (**b**) shows statistically significant differences only between the compressive strength values for PCM and the other composites with antibiotics. The remaining differences between all composites are described in the text above, with *p*-values specified. (*—the significance level (*p*-value) < 0.05).

Almost no statistically significant differences were observed between the compressive strength values for comparing all composites, including PCM, in dry conditions. The only statistically significant differences in dry conditions (*p* < 0.05) were observed between the compressive strength values for composite PCM/CIP0125/npCIP0125-CLI25 (No. 11) (a composite with two antibiotics incorporated in three ways—containing the highest amount of nanoparticles among all composites produced) and PCM/CIP0250-MET50-CLI50 (No. 8) (a composite with three antibiotics: two applied to the surface and one incorporated into the matrix; containing no nanoparticles) (Figure 7a).

Compressive stress (10% strain) values in wet conditions were obtained in the range of 0.00816–0.01160 MPa, which indicates an approximately 10-fold decrease in the compressive strength of the composites after soaking. The values were similar for the individual composite groups, as was the case in dry conditions. Similarly to the dry conditions, some statistically significant differences occurred within a group of composites with two antibiotics (surface-applied and incorporated into the PCM as an antibiotic solution) (Figure A3b). The PCM/CIP0125/npCIP0125-CLI25 composite exhibited the highest compressive strength of all composites (0.012 MPa) under wet conditions.

Notably, wetting of composites led to greater variability in compressive strength. Statistical analysis revealed a lack of similarity between the values of compressive strength of reference material—PCM and three composites: PCM-MET50-CLI50 (No. 1) (*p* = 0.025), PCM/CIP0250-MET25-CLI25 (No.7) (*p* = 0.036), and PCM/CIP0125/npCIP0125-CLI25 (No. 11) (*p* = 0.00013) (Figure 7b). Comparison of all composites under dry conditions revealed that the composite whose strength values differed statistically from those of all other composites was PCM/CIP0125/npCIP0125-CLI25 (No. 11) (*p* = 0.00013). Furthermore, statistically significant differences were also observed between the compressive strength values for: the PCM-MET50-CLI50 (No. 1) and all composites containing nanoparticles (0.00013 < *p* < 0.032); the PCM/CIP0250-CLI25 (No. 5) and composites containing nanoparticles (0.00013 < *p* < 0.019); and the PCM/CIP0250-MET25-CLI25 (No. 7) and all composites containing nanoparticles (0.00013 < *p* < 0.048).

Based on the obtained compressive strength values in dry and wet conditions, the strength retention (wet/dry compressive strength ratio) of the obtained composites was determined, as this indicates the maintenance of mechanical properties after wetting. PCM was used as the reference material, and the strength retention values of the obtained composites were compared with those of PCM. The obtained results indicate that, for five composites, the introduction of antibiotics decreased the retention of strength. This decrease was particularly noticeable in composites incorporating two antibiotics.

The obtained values for compressive stress in dry and wet conditions (for 10% strain) are presented in Figure 7.

**Figure A2:**
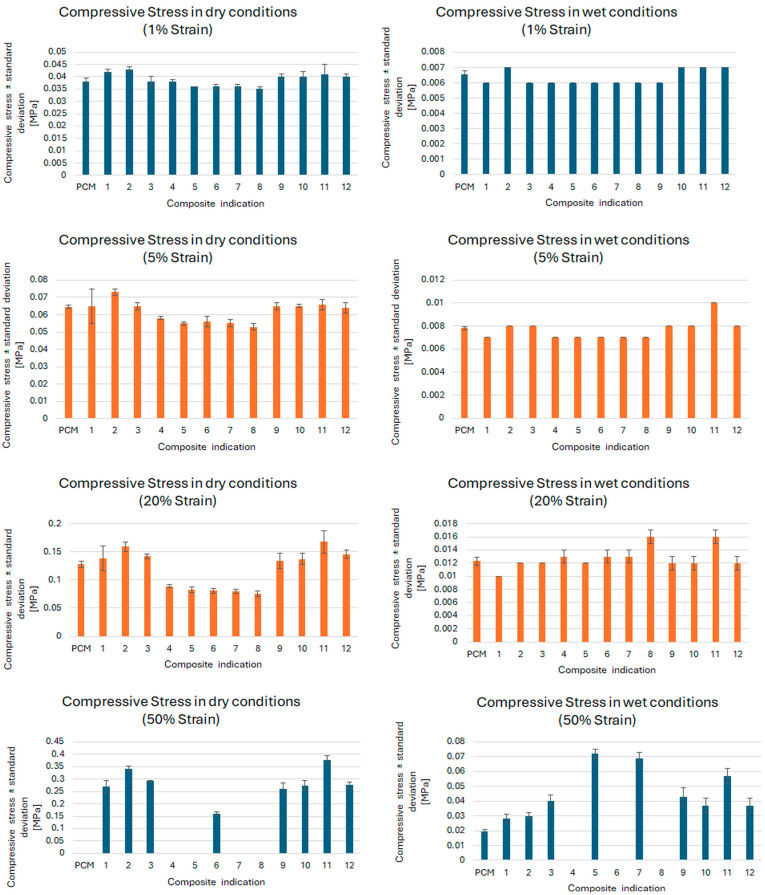
The values of compressive stress in dry and wet conditions (at 1–50% strain) for the obtained composites. Data represent four independent units of each composite (*n* = 4).

**Figure A3:**
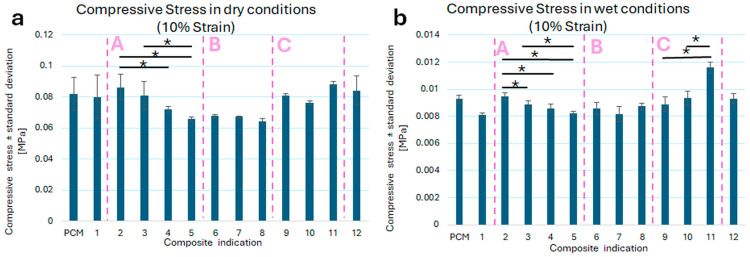
The values of compressive stress in (**a**) dry and (**b**) wet conditions (at 10% strain) for the obtained composites with statistically significant differences (*p* < 0.05) between values for individual composites inside a given group of composites (A—Composites with two antibiotics introduced: on the surface and inside the composite matrix in the antibiotic solution form, B—Composites with three antibiotics introduced: on the surface and inside the composite matrix in the antibiotic solution form, C—Composites with two antibiotics introduced: on the surface and inside the composite matrix in the antibiotic solution form and in the polymer nanoparticles form). Data represent four independent units of each composite (*n* = 4). (*—the significance level (*p*-value) < 0.05).

The obtained values of the composites’ compressive stress in dry and wet conditions for 1, 5, 20, and 50% strain are presented in Figure A2.

#### 3.2.4. Swelling Test

The aim of these studies was to determine the time required for the composite to reach equilibrium (a constant swelling ratio) and to determine the overall swelling degree of the resulting composite materials. The studies were conducted in 0.9% NaCl at pH 6.99, and at 37 °C for periods ranging from 1 h to 4 weeks.

**Figure A4:**
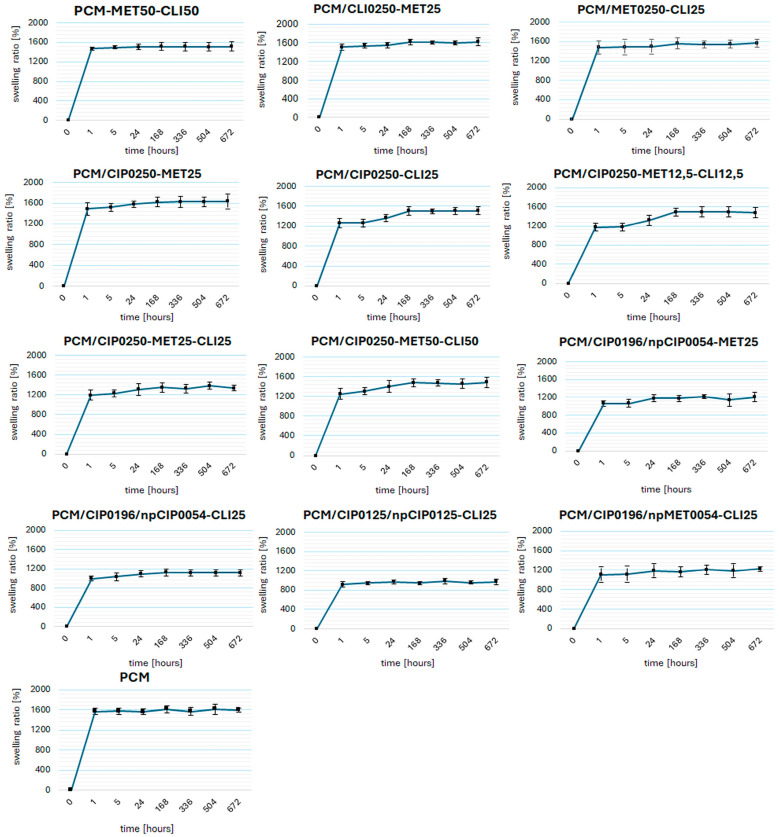
Swelling profiles of the composites with antibiotics determined during 672 h (4 weeks) of incubation in saline solution. Data represent five independent units of each composite (*n* = 5).

Figure A4 shows the obtained swelling profiles (swelling degree versus time) for individual composites. For all materials obtained, rapid liquid absorption is clearly observed within the first 60 min of immersion. This rapid absorption, along with a slight increase after 1 h, continues until swelling equilibrium is reached. The time to achieve equilibrium differs little for the individual composite types, but several distinct observations can be noted. Composites in which the antibiotic was incorporated into the polymer matrix in the form of a solution, during the composites’ synthesis, were characterized with approximately 168 h to reach the equilibrium swelling stage. Composites with antibiotics applied on the surface, and most composites in which antibiotics were additionally incorporated in the form of nanoparticles, reached the equilibrium swelling stage slightly faster. After reaching equilibrium, the swelling ratio remained constant for the four-week testing period.

**Figure 8:**
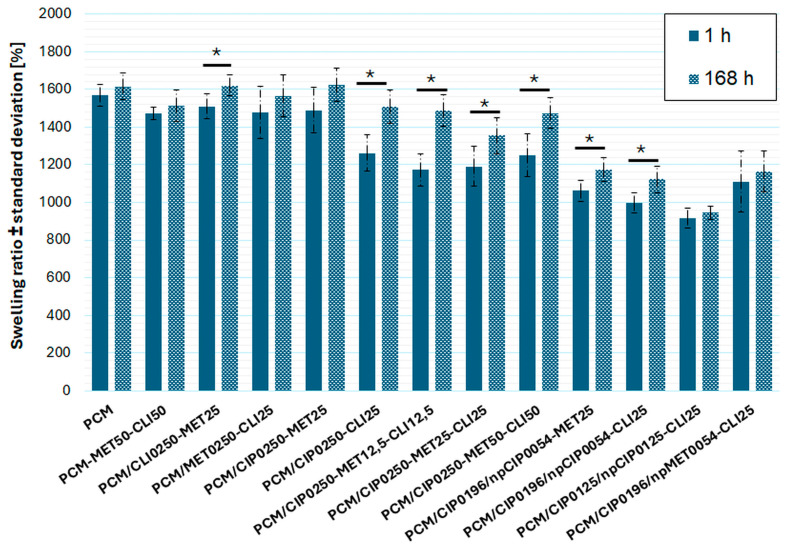
Swelling ratios of the composites with antibiotics after 1 h and 168 h of incubation in 0.9% NaCl with pH = 6.99, at 37 °C. Data represent five independent units of each composite (*n* = 5). (*—the significance level (*p*-value) < 0.05).

Figure 8 compares the swelling degrees of 12 individual composites with antibiotics and the blank carrier (PCM) after 1 h and 168 h of incubation (after reaching equilibrium swelling state for all composites). The overall swelling after 168 h of incubation of the resulting chitosan composites ranged from approximately 945% to 1624%, with the lowest values observed for composites containing nanoparticles (Table A3).

For individual composites statistically significant differences between swelling values after 1 h of incubation and after 168 h of incubation were observed: PCM/CLI0250-MET25 (*p* = 0.021), PCM/CIP0250-CLI25 (*p* = 0.0029), PCM/CIP0250-MET12.5-CLI12.5 (*p* = 0.00034), PCM/CIP0250-MET25-CLI25 (*p* = 0.034), PCM/CIP0250-MET50-CLI50 (*p* = 0.0068), PCM/CIP0196/npCIP0054-MET25 (*p* = 0.017) (Figure 8).

**Figure A5:**
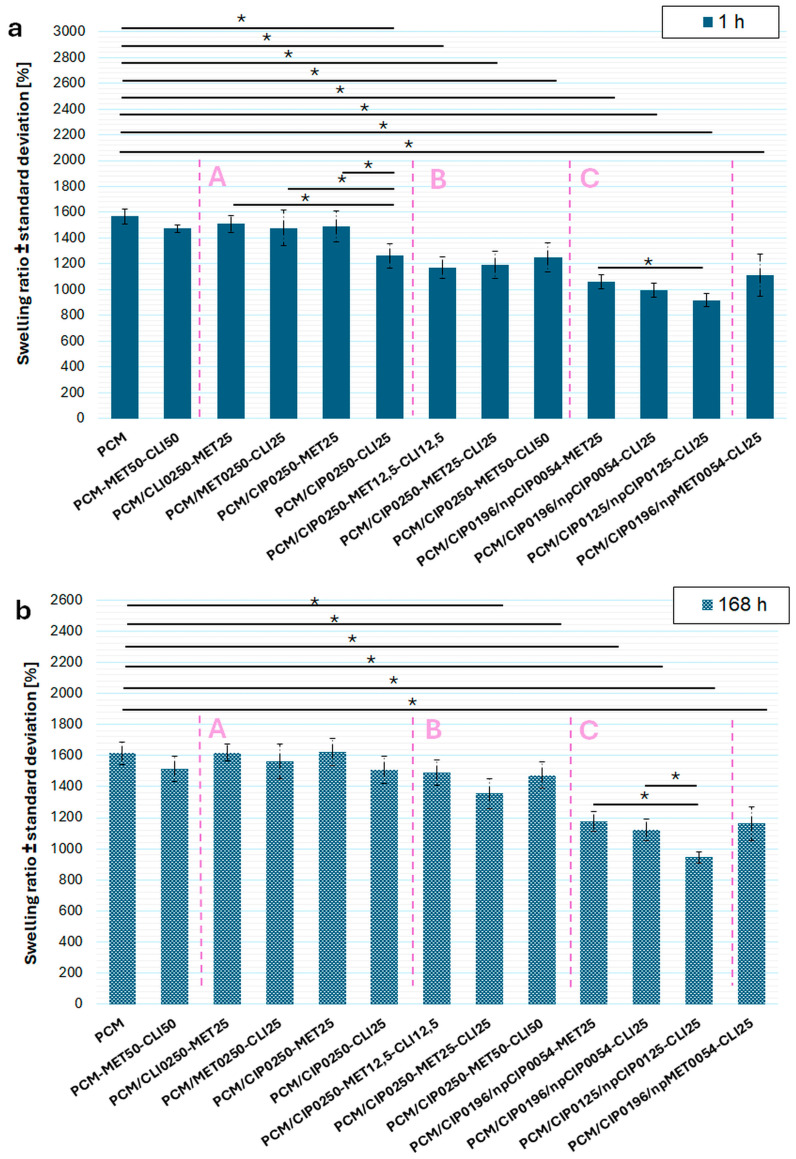
Swelling ratios of the composites with antibiotics: (**a**) after 1 h and (**b**) after 168 h of incubation in 0.9% NaCl with pH = 6.99, at 37 °C. Data represent five independent units of each composite (*n* = 5). Statistical analysis: statistically significant differences (*p* < 0.05) between swelling values of: PCM—blank carrier, and all composites with antibiotics; and individual composites inside a given group of composites (A—Composites with two antibiotics introduced: on the surface and inside the composite matrix in the antibiotic solution form, B—Composites with three antibiotics introduced: on the surface and inside the composite matrix in the antibiotic solution form, C—Composites with two antibiotics introduced: on the surface and inside the composite matrix in the antibiotic solution form and in the polymer nanoparticles form). (*—the significance level (*p*-value) < 0.05).

A comparative analysis of the blank carrier (PCM) and the obtained composites with antibiotics showed that the introduction of antibiotics into the composite matrix as nanoparticles resulted in statistically significant differences in swelling values measured after 1 h and 168 h of incubation. Additionally, statistically significant differences in swelling values measured after 1 h of incubation were observed between PCM and four composites with antibiotics introduced on the surface and within the composite matrix in antibiotic solution form (Figure A5a,b).

### 3.3. Antibiotics Release from Composites

**Figure 9:**
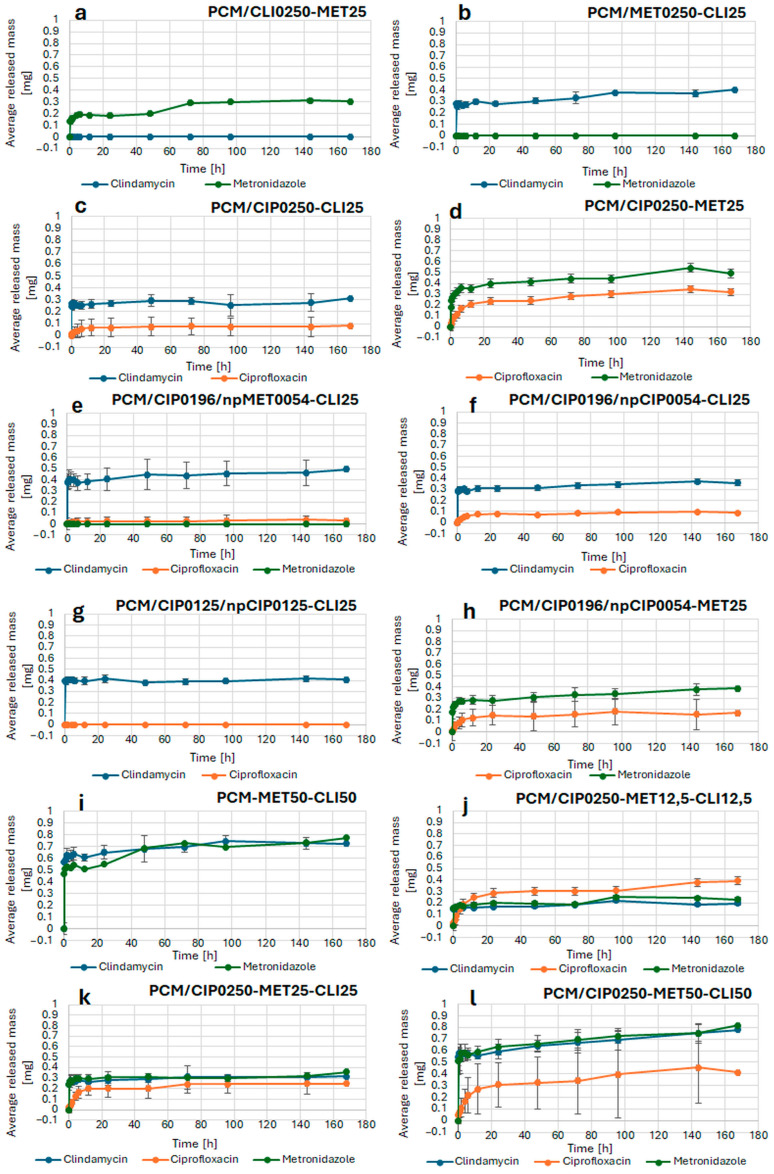
Drug release profiles obtained using the basket dissolution apparatus in 0.9% NaCl as the release medium for 168 h; from composite: (**a**) PCM/CLI0250-MET25; (**b**) PCM/MET0250-CLI25; (**c**) PCM/CIP0250-CLI25; (**d**) PCM/CIP0250-MET25; (**e**) PCM/CIP0196/npMET0054-CLI25; (**f**) PCM/CIP0196/npCIP0054-CLI25; (**g**) PCM/CIP0125/npCIP0125-CLI25; (**h**) PCM/CIP0196/npCIP0054-MET25; (**i**) PCM-MET50-CLI50; (**j**) PCM/CIP0250-MET12,5-CLI12,5; (**k**) PCM/CIP0250-MET25-CLI25; (**l**) PCM/CIP0250-MET50-CLI50. Data represent three independent units of each composite (*n* = 3).

Figure 9 presents the release profiles of the active substances from the composites obtained. Because the strategy of incorporating active substances was closely linked to the release results, the graph order in the figure is consistent with the considerations below.

To determine which methods of incorporation for each antibiotic were effective for release, two active substances were first incorporated using two methods: application to the composite surface and incorporation into the polymer matrix. Release experiments revealed that for the PCM/CLI0250-MET25 (Figure 9a) and PCM/MET0250-CLI25 (Figure 9b) composites, the concentrations of clindamycin and metronidazole incorporated into the matrix remained below the limit of quantification (LOQ), indicating no detectable release under the experimental conditions used. The LOQ values were 19 µg/g for clindamycin and 188 µg/g for metronidazole. The release of metronidazole and clindamycin from the surface of these composites was observed. The most favorable release profiles were obtained for the composite PCM/CIP0250-MET25 (Figure 9d), in which ciprofloxacin was incorporated into the matrix, and metronidazole was incorporated on the surface.

To obtain a formulation containing three antibiotics, metronidazole encapsulated within nanoparticles was additionally incorporated into the composite with ciprofloxacin incorporated into the matrix and clindamycin applied on the surface. However, release results from the resulting composite PCM/CIP0196/npMET0054-CLI25 (Figure 9e) showed that metronidazole was not released from the polymer nanoparticles. Therefore, it was concluded that metronidazole could be effectively incorporated only by applying it to the composite surface.

In the next step, it was examined whether the introduction of ciprofloxacin not only into the composite matrix but also its encapsulation in polymer nanoparticles would affect the release profile. The considered composites, PCM/CIP0196/npCIP0054-CLI25 (Figure 9f), PCM/CIP0196/npCIP0054-MET25 (Figure 9h), and PCM/CIP0125/npCIP0125-CLI25 (Figure 9g), contained two active substances. Ciprofloxacin was incorporated into a polymer matrix both as the antibiotic solution form and as nanoparticles, whereas metronidazole or clindamycin was applied on the composite surface. Release data analysis confirmed that clindamycin and metronidazole were released from the composite surface, consistent with observations for PCM/CLI0250-MET25 (Figure 9a), PCM/CIP0250-MET25 (Figure 9d), PCM/CIP0250-CLI25 (Figure 9c), and PCM/MET0250-CLI25 (Figure 9b). In contrast, encapsulation of ciprofloxacin in polymer nanoparticles did not confer a beneficial effect on its release. Increasing the fraction of ciprofloxacin in nanoparticles at the expense of its content introduced into the matrix in the antibiotic solution form resulted in lower amounts of ciprofloxacin released, as observed for PCM/CIP0196/npCIP0054-CLI25 (Figure 9f) compared with PCM/CIP0125/npCIP0125-CLI25 (Figure 9g).

Based on the obtained results, it was subsequently examined whether co-applying two antibiotics—metronidazole and clindamycin, according to earlier findings—on the composite surface, and thereby effectively incorporated, would alter their release kinetics. To this end, the PCM-MET50-CLI50 (Figure 9i) composite was fabricated, with both active substances deposited on the surface. Statistical comparison using similarity and difference factors (f_2_ and f_1_) demonstrated equivalence of the clindamycin release profiles for PCM/CIP0196/npCIP0054-CLI25 (Figure 9f) and PCM-MET50-CLI50 (Figure 9i), as well as equivalence of the metronidazole release profiles for PCM/CIP0196/npCIP0054-MET25 (Figure 9h) and PCM-MET50-CLI50 (Figure 9i). Collectively, these results indicate that co-applying metronidazole and clindamycin on the surface does not adversely affect their release profiles.

In the context of the above observations, the release was examined in the following composites: PCM/CIP0250-MET12.5-CLI12.5 (Figure 9j), PCM/CIP0250-MET25-CLI25 (Figure 9k) and PCM/CIP0250-MET50-CLI50 (Figure 9l), in which clindamycin and metronidazole were introduced onto the composite surface in different amounts, while ciprofloxacin was introduced into the matrix at an identical dose in all formulations. In PCM/CIP0250-MET12.5-CLI12.5 (Figure 9j), the surface-loaded antibiotic content was the lowest, whereas in the subsequent composites it was increased twofold. Release studies demonstrated efficient release of all active substances from each composite. However, statistical evaluation using similarity and difference factors (f_2_ and f_1_) revealed a lack of similarity between the release profiles of the investigated formulations. Notably, increasing the amount of clindamycin and metronidazole applied on the surface led to greater variability in ciprofloxacin release between individual dosage units. The most favorable release characteristics, along with a balanced antibiotic ratio, were observed for PCM/CIP0250-MET12.5-CLI12.5 (Figure 9j) and PCM/CIP0250-MET25-CLI25 (Figure 9k). These composites exhibited rapid release of substantial amounts of the active substances within a short time frame.

## 4. Discussion

Three types of multi-stage procedures were developed for the simultaneous introduction of antibiotics: ciprofloxacin, metronidazole, and clindamycin into the porous composite systems, which could ensure their synergistic effect in the intended use of the resulting composite carrier. Considering that an effective drug carrier should have an appropriate release profile, ensuring local, immediate, and yet sustained release of these active substances, three types of composites with two or three antibiotics incorporated in different ways were designed for comparison. One of the best ways to prolong drug release from a hydrogel is to introduce an additional carrier, such as micro- or nanocapsules, into the hydrogel matrix [7]. PLGA-based nanocapsules were developed and obtained in this work.

In general, controlled drug-delivery systems with diameters ranging up to 1000 nm and a polymer core can significantly enhance the biopharmaceutical properties of antibacterial agents [29]. The size of the nanoparticles obtained with both ciprofloxacin and metronidazole exceeds 50 nm, which, according to the literature [9,30], should prolong the release of the active substance. The zeta potential (ζ) of the synthesized nps serves as a critical indicator of surface charge, fundamentally influencing both colloidal stability and antimicrobial behavior. In this study, the obtained nps exhibited a negative zeta potential in both media—1% acetic acid and water. This charge can be attributed to the terminal carboxyl group of PLGA. The absolute Zeta potential values obtained in water and in acetic acid indicate better particle stabilization in the acid. While a high absolute zeta potential is often associated with enhanced electrostatic interplay (greater nanoparticle stability), the interaction between these negatively charged nps and the similarly charged bacterial cell walls may likely involve localized drug release or the induction of reactive oxygen species (ROS) rather than simple attraction [30,31]. The low entrapment of CIP and MET in nanoparticles may be due to drug diffusion during the secondary emulsion and solvent evaporation steps. The solubility of both APIs—CIP and MET—was investigated experimentally, and it was found that API CIP is more than 20 times more soluble than API MET. Therefore, during evaporation, excess CIP can interact with the aqueous phase, reducing drug entrapment by nanoparticles. Optimization of the nanoparticles was performed for both types, but only a limited number of parameters were tested (e.g., PVA concentration at three levels). However, the literature [32,33] indicates that each parameter also affects the other, and no clear conclusions can be drawn. Therefore, further optimization would be necessary.

In the analyzed systems, release kinetics are strictly correlated with nanoparticle architecture; for nanospheres with a uniformly dispersed payload, release occurs via a combination of matrix erosion and diffusion [34]. Drugs with relatively low molecular weight (<600 Da), such as ciprofloxacin and metronidazole, usually exhibit a biphasic release profile. While literature suggests that the initial burst effect typically arises from a drug fraction weakly adsorbed onto the large specific surface area of the nps, this effect was effectively minimized in the present study. Typical release profiles for drug-loaded PLGA nps show early release of a large proportion of the drug—for both drugs, after 4 days, almost 39% of the ciprofloxacin and almost 53% of the metronidazole had been released over the entire study period (28 days—672 h). During the first 24 h, ciprofloxacin release was negligible, attributed to limited surface adsorption and the initial swelling of the PLGA matrix, which temporarily restricted pore access. The PLGA 50:50 copolymer utilized here, being an amorphous material, undergoes bulk erosion more rapidly than variants with higher lactide content. Incubation in 0.9% NaCl induces ester bond hydrolysis, reducing the polymer’s molecular weight and increasing its polydispersity. This chain-degradation process facilitated drug diffusion into the external medium, with metronidazole eluting faster than ciprofloxacin [35]. The drug content, based on drug loading (DL), was 0.17 µg for ciprofloxacin and 1.69 µg for metronidazole per milligram of carrier.

Regarding the developed antibiotic carriers (with and without nanoparticles), the physicochemical properties of the composites (microstructure, mechanical strength, and swelling) depend on both the material composition and the production method.

The porous microstructure of composites obtained by lyophilization through freezing and solvent sublimation generally depends on the concentration of the suspension undergoing this process, the amount and grain size of the filler, and the method of its incorporation [36]. The results suggest that introducing a BaSO_4_ additive into the chitosan/bioglass system still allows the production of sponges with evenly distributed, open pores throughout the volume, while also enabling radiological contrast [37]. Similarly, the addition of antibiotics does not significantly alter the porous microstructure. A higher concentration of smaller grains in the pore walls of composites with antibiotics introduced in solution, compared to those introduced on the surface, may be due to some precipitation of antibiotics during synthesis, giving the impression of additional filler grains. The observed differences in filler arrangement are reflected in the pore-size distribution: composites with ciprofloxacin introduced in antibiotic solution form into the polymer matrix during synthesis exhibit a predominant number of pores in the larger range (40–60 µm), whereas the other composites considered (20–40 µm) have a smaller number of pores. Despite differences in pore sizes across individual composites in the statistical analysis, the narrow pore-size distribution in the histograms (Figure 6) suggests that the pores within a given composite are similar in size. Therefore, the drug’s diffusion from the interior of the material will occur at a similar rate throughout its volume, which is of great importance for clinical interpretation.

The microstructure of porous composites also influences their mechanical properties, particularly in compressive strength tests conducted on dry samples. Composites with a predominant number of pores in the 40–60 µm range have an average compressive strength of approximately 18% lower than the other composites considered. This can be justified by the observation that a larger number of small pores increases the mechanical strength of the system more effectively than a smaller number of larger pores, while maintaining the same component concentrations. Increasing the effective load-bearing surface can improve mechanical properties [38]. The highest mechanical strength was achieved by the PCM/CIP0125/npCIP0125-CLI25 composite, in which the antibiotic was incorporated at the highest loading as nanoparticles. Considering that the nanoparticle shell is composed of PLGA, this is an obvious result. The addition of nanoparticles composed of a higher-strength material strengthens the chitosan matrix. The mechanical strength of composites in their dry state is almost an order of magnitude greater than that of composites soaked in saline solution. This is due to liquid absorption, the penetration of the liquid medium between polymer chains, and the system’s elastification. Nevertheless, determining the wet mechanical strength and the strength retention (wet/dry compressive strength ratio) was essential for preparation and for assessing the system’s integrity under conditions similar to those used; a higher wet/dry strength retention indicates better maintenance of mechanical strength during clinical handling.

In the intended clinical application, the composite will be placed directly onto the exposed pulp in several small increments to ensure intimate adaptation to the pulp chamber morphology. After placement, the material will be moistened with sterile 0.9% NaCl solution to provide appropriate conditions for the diffusion of the active substances. The tooth will then be sealed with a temporary restoration. After the required treatment period, the composite will be completely removed from the pulp chamber. Owing to its cohesive structure and mechanical stability, the material does not disintegrate in situ and therefore can be removed without leaving residual particles. This represents an important practical advantage over paste-based pulp medicaments, which are often difficult to remove completely and may leave remnants on dentinal surfaces or within dentinal tubules.

The intended application of the carriers being developed is periodontal treatment for tooth root diseases, in which the composites will release drugs upon contact with a saline solution. Since the rate and profile of antibiotic release from hydrogels are primarily influenced by liquid absorption and diffusion, the swelling degree of the obtained multi-antibiotic porous composites was evaluated by incubation in 0.9% NaCl. The obtained swelling test results for the composites indicate rapid liquid absorption within the first 60 min of incubation in 0.9% NaCl and high swelling rates, most likely resulting from the presence of hydrophilic groups in the chitosan chains and the high porosity of the material, which facilitates the diffusion of water molecules into the interior of the material [7]. The longer time required to reach swelling equilibrium for composites containing antibiotics in PCM, compared with other composites, can be attributed to their microstructure. Composites with larger pores most likely have a smaller internal surface area through which liquid diffusion occurs; hence, swelling equilibrium is achieved somewhat later. From a clinical application perspective, statistically significant differences in swelling values between 1 h and 168 h of incubation for these composites are an advantage. They indicate a longer swelling time to equilibrium after the initial rapid swelling due to the absorption of a significant amount of medium. The longer the swelling time, the longer the release of the active substance. These considerations suggest that drug release from such systems will occur immediately and be sustained over a longer period. The absence of mass loss observed after achieving swelling equilibrium for up to four weeks of testing suggests that the carrier’s polymer matrix does not degrade during incubation, meaning that in clinical use, this will allow the material to be used for the intended treatment time and then removed without leaving residual particles.

The effectiveness of hydrogel drug carriers depends on their physicochemical properties, the methods and mechanisms of drug loading and release, and the properties of the active substances introduced. Typically, loaded hydrogel carriers obtained by directly delivering (permeating) the active substance to previously cross-linked hydrogels are characterized by a rapid, explosive release profile. Entrapment of the drug in the hydrogel matrix due to cross-linking of hydrogel chains in the presence of the loaded drug may yield better results, but only to a limited extent. The main mechanisms limiting release in this case are diffusion and swelling. Meanwhile, sustained release occurs for some drugs encapsulated in the gel network and depends on the degree of hydrogel cross-linking [7,39].

In the composite carriers we developed, the most important observation was that metronidazole and clindamycin incorporated into the composite matrix during synthesis were not ultimately released from the finished composites, whereas the release profile of ciprofloxacin incorporated in this manner indicated its effective release.

Generally, several different mechanisms may be responsible for the lack of antibiotic release from a matrix. The first cause may be limited diffusion due to insufficient porosity in the dense polymer network, low solubility of the active substance in the polymer, or insufficient swelling of the matrix in the release medium. Studies show that the porosity, hydrophilicity, and compactness of the polymer matrix strongly influence the migration of active substances. Increased porosity and hydrophilicity lead to faster release by lowering the diffusion barrier [40]. The second cause of the lack of antibiotic release from the matrix may be strong drug-polymer interactions (hydrogen bonds, adsorption, complexation), which can inhibit the migration of the substance from the matrix. In such a situation, the drug is permanently physicochemically bound and does not migrate into the release medium, thus failing to reach a measurable concentration in the release medium [41]. Drug interactions with the polymer phase are a key mechanism regulating release [42]. Another known reason for limiting antibiotic release from the matrix is insufficient matrix degradation/erosion, or a slow rate. Polymer bioerosion models show that polymer degradation and pore formation (new diffusion channels) are the main factors controlling release kinetics [43]. A lack of or limited release can also be caused by the recrystallization or aggregation of the active substance in the matrix, which reduces its mobility and release rate [44]. Another cause of limited release may be uneven distribution of the active substance in the matrix. In such a case, diffusion occurs only from drug-rich areas, and if these areas are located deep, release is undetectable in the first hours/days [45]. Finally, drug release does not occur if the drug undergoes chemical degradation and loses its activity, resulting in an apparent lack of a release profile. Reviews of controlled-release systems emphasize the influence of composition, processing conditions, and drug-polymer compatibility on matrix stability [46].

The lack of detectable release (<LOQ) from the matrix for PCM/CLI0250-MET25 and PCM/MET0250-CLI25 may be due to a combination of several of the above phenomena: limited diffusion, strong drug-matrix interactions, insufficient polymer degradation, crystallization, or aggregation of substances in the matrix. The fact that the same substances were released without issue after application to the surface indicates that the mechanism of limitation relates solely to migration from the polymer matrix interior, not to analytical limitations of the method or to drug stability in solution. The reason for these observations may also be that metronidazole and clindamycin, being more hydrophilic than ciprofloxacin and soluble in an aqueous environment, could have been washed out of the carrier during stabilization. This indicates that the proposed method of introduction via entrapment in the polymer matrix is unsuitable for these two antibiotics in our developed composites. However, the second method (which involves applying the antibiotic to the composite surface and allowing the previously stabilized PCM to be penetrated by the antibiotic solution) proved to be an effective way to introduce metronidazole and clindamycin into the developed composite chitosan carriers. The release profiles of the antibiotics introduced in this manner showed rapid, constant release throughout the experiment, and the surface co-application of metronidazole and clindamycin did not adversely affect their release profiles in the resulting composites.

The resulting release profile is advantageous compared to other formulations used by dentists, especially those that release the drug immediately upon contact with water. In such cases, it is impossible to control the kinetics of drug release [47], and only a small portion of the dose reaches the target site, with the majority being distributed to other tissues and metabolized or excreted. In dental applications, where salivary flow and swallowing movements rapidly lead to drug dissolution and elimination, maintaining an effective drug concentration on infected surfaces and target tissues is crucial. For this reason, drug-delivery systems (DDS) are being developed, thanks to which drugs are released in a controlled manner at the target site, which often minimizes their overall distribution in the body and minimizes the risk of side effects such as systemic toxicity [48,49,50]. The developed composite system is also designed for target-site application, thus ensuring improved drug efficacy for the intended use.

Release studies for the developed composite drug carrier system were conducted in 0.9% NaCl, pH = 6.99, as the release medium intended for clinical use. Given that pH can be lower in teeth with periodontal disease, the composite carrier was designed using chitosan. For this polymer, pH stimulation is a known pathway for controlled release. Cationic chitosan (one of the few positively charged natural polymers) reacts at low pH, inducing swelling and dissolution, which facilitates the release of the active substance [7]. Furthermore, the solubility of some of the drugs used is pH-dependent, which could affect release behavior. It is known from the literature that antibiotics such as amoxicillin and metronidazole showed good release from hydrogel swelling in enzyme-free simulated gastric fluid (pH 1.2), allowing the release of most of the amoxicillin (65%) and metronidazole (59%). In contrast, in simulated intestinal fluid (pH 7.2), they showed limited release after 2 h [51].

The obtained composites release active substances rapidly during the initial stage, allowing therapeutic antibiotic concentrations to be reached quickly at the site of application. Importantly, the concentrations achieved locally exceed the minimum inhibitory concentration (MIC) for relevant oral microorganisms, including Enterococcus faecalis [52], Fusobacterium nucleatum [53,54], Streptococcus mutans [55], Streptococcus oralis [53], and Porphyromonas gingivalis [54,55]. The formulation contains antibiotics commonly used to treat oral and dental infections, namely metronidazole, clindamycin, and ciprofloxacin. Maintaining antibiotic levels above the MIC at the infection site is advantageous, as it may reduce the risk of microbial resistance development, which represents a significant benefit of this dosage form.

An interesting observation was the lack of metronidazole release from composites containing this antibiotic as nanoparticles embedded in a polymer matrix, and the finding that increasing the ciprofloxacin fraction in the nanoparticles, at the expense of its content in the matrix, also decreased the amount of ciprofloxacin released. This could be explained by the fact that the release of the active substance from nanoparticles occurs through a combination of matrix erosion and diffusion [34]. In the case of nanoparticles embedded in a matrix, there is likely an additional stage of erosion, diffusion, or degradation of the hydrogel matrix. Moreover, as measured early, nanoparticles loaded with ciprofloxacin showed negligible release during the first experimental period (24 h), followed by very low release thereafter. Given the demonstrated lack of hydrogel matrix degradation over four weeks, it is understandable that there is no visible release of antibiotics from the developed composite matrix in the form of nanoparticles. However, the values of ciprofloxacin and metronidazole released from nanoparticles (µg of released antibiotics—Table A1) and MIC values mentioned in this article in the earlier paragraph indicate that, despite the small amount of antibiotics enclosed in nanoparticles, they could potentially exhibit their own or improved composite antibacterial activity.

Since the rate and profile of antibiotic release are key factors determining the therapeutic effect in the intended application, a ranking list of the obtained composites was created based on the release results (Table 4).

## 5. Conclusions

In our work, we developed and characterized multi-antibiotic porous composites based on chitosan, bioglass, and BaSO_4_, which could be a tailored drug-delivery system in dentistry. We developed a strategy for introducing three antibiotics recommended by the American Academy of Pediatric Dentistry as components of a three-antibiotic paste used in periodontology for the treatment of tooth root diseases: ciprofloxacin, metronidazole, and clindamycin. We examined the release profiles of these antibiotics. We demonstrated that, in porous chitosan/bioglass/BaSO_4_ systems, the most advantageous method for introducing metronidazole and clindamycin, in terms of release profile, is to apply them to the surface of the stabilized PCM. Ciprofloxacin, by contrast, can be incorporated directly into the polymer matrix and entrapped within the matrix during synthesis of composites. These composites exhibited rapid release of substantial amounts of the active substances within a short time frame and maintained their concentration for an extended period. Such release behavior is considered advantageous for the treatment of microbial infections, as it may facilitate rapid reduction in microbial burden.

## Figures and Tables

**Table 1:** Porous composites with antibiotics introduced by three different methods.

No.	Name of Composite	Antibiotic Applied on the Surface of Porous Composite [Name/Amount Introduced per 1 g of PCM/Concentration of Antibiotic Solution Used]	Antibiotic Incorporated in the Composite Matrix	
			in the Antibiotic Solution Form [Name/Amount Introduced per 1 g of PCM/Concentration of Antibiotic Solution Used]	in the Polymer Nanoparticles Form [Name/Amount Introduced per 1 g of PCM/Concentration of Antibiotic Solution Used]
**Composite with two antibiotics introduced (on the surface of PCM)**				
**1**	***PCM-MET50-CLI50***	metronidazole + clindamycin/50 mg + 50 mg/2.5 mg·mL^−1^ +2.5 mg·mL^−1^		
**Composites with two antibiotics introduced (on the surface and inside the matrix of PCM in the antibiotic solution form)**				
**2**	***PCM/CLI0250-MET25***	metronidazole/31.28 mg/1.25 mg·mL^−1^	clindamycin/251.04 mg/50.35 mg·mL^−1^	
**3**	***PCM/MET0250-CLI25***	clindamycin/31.32 mg/1.25 mg·mL^−1^	metronidazole/252.70 mg/13.08 mg·mL^−1^	
**4**	***PCM/CIP0250-MET25***	metronidazole/31.34 mg/1.25 mg·mL^−1^	ciprofloxacin/253.61 mg/12.02 mg·mL^−1^	
**5**	***PCM/CIP0250-CLI25***	clindamycin/31.25 mg/1.25 mg·mL^−1^	ciprofloxacin/250.00 mg/11.81 mg·mL^−1^	
**Composites with three antibiotics introduced (on the surface and inside the matrix of PCM in the antibiotic solution form)**				
**6**	***PCM/CIP0250-MET12,5-CLI12,5***	metronidazole + clindamycin/15.63 mg + 15.63 mg/0.625 mg·mL^−1^ + 0.625 mg·mL^−1^	ciprofloxacin/250.00 mg/11.81 mg·mL^−1^	
**7**	***PCM/CIP0250-MET25-CLI25***	metronidazole + clindamycin/31.25 mg + 31.25 mg/1.25 mg·mL^−1^ + 1.25 mg·mL^−1^	ciprofloxacin/250.00 mg/11.81 mg·mL^−1^	
**8**	***PCM/CIP0250-MET50-CLI50***	metronidazole + clindamycin/62.50 mg + 62.50 mg/2.5 mg·mL^−1^ + 2.5 mg·mL^−1^	ciprofloxacin/250.00 mg/11.81 mg·mL^−1^	
**Composites with two antibiotics introduced (on the surface and inside the matrix of PCM in the antibiotic solution form and in the polymer nanoparticles form)**				
**9**	***PCM/CIP0196/npCIP0054-MET25***	metronidazole/35.95 mg/1.25 mg·mL^−1^	ciprofloxacin/195.86 mg/10.05 mg·mL^−1^	ciprofloxacin/1.00 mg/0.05 mg·mL^−1^
**10**	***PCM/CIP0196/npCIP0054-CLI25***	clindamycin/35.95 mg/1.25 mg·mL^−1^	ciprofloxacin/195.86 mg/10.05 mg·mL^−1^	ciprofloxacin/1.00 mg/0.05 mg·mL^−1^
**11**	***PCM/CIP0125/npCIP0125-CLI25***	clindamycin/44.06 mg/1.25 mg·mL^−1^	ciprofloxacin/125.12 mg/6.41 mg·mL^−1^	ciprofloxacin/1.00 mg/0.05 mg·mL^−1^
**Composite with three antibiotics introduced (on the surface and inside the matrix of PCM in the antibiotic solution form and in the polymer nanoparticles form)**				
**12**	***PCM/CIP0196/npMET0054-CLI25***	clindamycin/36.77 mg/1.25 mg·mL^−1^	ciprofloxacin/195.88 mg/10.05 mg·mL^−1^	metronidazole/8.00 mg/0.41 mg·mL^−1^

**Table 2:** Properties of obtained PLGA-antibiotic nanoparticles (mean ± std. dev.).

		npCIP	npMET
DLS/H_2_O	Zeta potential ± std. dev. [mV]	−34.8 ± 1.7	−38.5 ± 2.9
	Z-Ave ± std. dev. [d.nm]	1280 ± 409	1743 ± 268
	PDI ± std. dev.	0.85 ± 0.14	0.96 ± 0.07
	D(v) 0.1 ± std. dev. [nm]	200 ± 42	169 ± 65
	D(v) 0.5 ± std. dev. [nm]	247 ± 54	262 ± 53
	D(v) 0.9 ± std. dev. [nm]	305 ± 70	329 ± 83
DLS/1% CH_3_COOH	Zeta potential ± std. dev. [mV]	−1.7 ± 0.5	−3.3 ± 0.2
	Z-Ave ± std. dev. [d.nm]	764 ± 331	585 ± 166
	PDI ± std. dev.	0.66 ± 0.16	0.66 ± 0.11
	D(v) 0.1 ± std. dev. [nm]	243 ± 101	169 ± 57
	D(v) 0.5 ± std. dev. [nm]	249 ± 140	185 ± 75
	D(v) 0.9 ± std. dev. [nm]	374 ± 124	322 ± 36
UV-vis	DL ± std. dev. [%]	0.10 ± 0.02	0.76 ± 0.10
	EE ± std. dev. [%]	0.60 ± 0.10	3.41 ± 0.46

**Table 3:** The values of compressive stress at 10% strain for the obtained composites (the values were presented with standard deviation), and the strength retention. Data represent four independent units of each composite (*n* = 4).

No.	Name of Composite	Compressive Stress (10% Strain) in Dry Condition [MPa]	Compressive Stress (10% Strain) in Wet Condition [MPa]	Strength Retention [%]
**Blank carrier**				
0	PCM	0.082 ± 0.011	0.00931 ± 0.00030	11.35
**Composite with two antibiotics introduced (on the surface of PCM)**				
1	PCM-MET50-CLI50	0.080 ± 0.014	0.00809 ± 0.00015	10.11
**Composites with two antibiotics introduced (on the surface and inside the matrix of PCM in the antibiotic solution form)**				
2	PCM/CLI0250-MET25	0.0862 ± 0.0083	0.00949 ± 0.00028	11.01
3	PCM/MET0250-CLI25	0.0810 ± 0.0091	0.00889 ± 0.00026	10.98
4	PCM/CIP0250-MET25	0.0719 ± 0.0018	0.00860 ± 0.00031	11.96
5	PCM/CIP0250-CLI25	0.0656 ± 0.0016	0.00824 ± 0.00015	12.56
**Composites with three antibiotics introduced (on the surface and inside the matrix of PCM in the antibiotic solution form)**				
6	PCM/CIP0250-MET12,5-CLI12,5	0.0676 ± 0.0012	0.00857 ± 0.00046	12.68
7	PCM/CIP0250-MET25-CLI25	0.06706 ± 0.00063	0.00816 ± 0.00057	12.17
8	PCM/CIP0250-MET50-CLI50	0.0642 ± 0.0020	0.00874 ± 0.00024	13.62
**Composites with two antibiotics introduced (on the surface and inside the matrix of PCM in the antibiotic solution form and in the polymer nanoparticles form)**				
9	PCM/CIP0196/npCIP0054-MET25	0.081 ± 0.011	0.00889 ± 0.00054	10.96
10	PCM/CIP0196/npCIP0054-CLI25	0.076 ± 0.012	0.00934 ± 0.00053	12.29
11	PCM/CIP0125/npCIP0125-CLI25	0.088 ± 0.019	0.01160 ± 0.00041	13.18
**Composite with three antibiotics introduced(on the surface and inside the matrix of PCM in the antibiotic solution form and in the polymer nanoparticles form)**				
12	PCM/CIP0196/npMET0054-CLI25	0.0842 ± 0.0095	0.00931 ± 0.00036	11.06

**Table 4:** Comparing the obtained multi-antibiotic porous systems by simply scoring the most favorable and least favorable in terms of release efficiency.

Ranking	Name of Composite	Comment
1	PCM/CIP0250-MET12.5-CLI12.5	The most favorable release characteristics, along with a balanced antibiotic ratio.
2	PCM/CIP0250-MET25-CLI25	The most favorable release characteristics, along with a balanced antibiotic ratio.
3	PCM/CIP0250-MET50-CLI50	The highest average released mass and favorable release characteristics, but the results are subject to large errors.
4	PCM-MET50-CLI50	The result of the average released mass is very high, but in the intended use, the composite should contain three antibiotics.
5	PCM/CIP0250-MET25	The best result of average released mass and the most favorable release characteristics among composites with two antibiotics, one of which is incorporated into the mass.
6	PCM/CIP0196/npCIP0054-MET25	The average released mass is lower than that of the above composites, and in the intended use, the composite should contain three antibiotics.
7	PCM/CIP0196/npCIP0054-CLI25	The average released mass is lower than that of the above composites, and in the intended use, the composite should contain three antibiotics.
8	PCM/CIP0250-CLI25	The average released mass is lower than that of the above composites, and in the intended use, the composite should contain three antibiotics.
9	PCM/CIP0125/npCIP0125-CLI25	Introducing more antibiotics in the form of nanoparticles further reduces the average released mass, and, in the intended use, the composite should contain three antibiotics.
10	PCM/CIP0196/npMET0054-CLI25	Despite the high release value of clindamycin, the other two antibiotics introduced into the composite are released only to a small extent.
11	PCM/MET0250-CLI25	The concentrations of metronidazole incorporated into the matrix remained below the limit of quantification (LOQ).
12	PCM/CLI0250-MET25	The concentrations of clindamycin incorporated into the matrix remained below the limit of quantification (LOQ).

## Data Availability

The original contributions presented in this study are included in the article. Further inquiries can be directed to the corresponding author.

## Abbreviations

PCM	porous composite matrix
CIP	ciprofloxacin
MET	metronidazole
CLI	clindamycin

## Appendix A

**Table A1:** Release data of ciprofloxacin and metronidazole (mean and standard deviation from 3 replicates). Data are shown as µg of released antibiotic, µg of released antibiotic/1 mg of nanoparticles, and percentage released rate.

Time [days]	Time [h]	npCIP			npMET		
		Mean ± Std. Dev. [µg]	Mean ± Std. Dev. [µg/mg]	Mean ± Std. Dev. [%]	Mean ± Std. Dev. [µg]	Mean ± Std. Dev. [µg/mg]	Mean ± Std. Dev. ± Std. dev. [%]
0	0	0	0	0	0	0	0
0.04	1	0.00 ± 0.00	0.00 ± 0.00	0.0 ± 0.0	0.79 ± 0.63	0.06 ± 0.05	3.7 ± 3.0
0.13	3	0.00 ± 0.00	0.00 ± 0.00	0.0 ± 0.0	1.38 ± 0.51	0.11 ± 0.04	6.5 ± 2.4
1.00	24	0.02 ± 0.06	0.00 ± 0.00	1.0 ± 2.8	6.0 ± 1.46	0.48 ± 0.19	28.3 ± 11.1
2.00	48	0.08 ± 0.10	0.01 ± 0.01	3.9 ± 4.9	6.01 ± 0.02	0.48 ± 0.00	28.3 ± 0.1
4.00	96	0.61 ± 0.21	0.05 ± 0.02	28.8 ± 9.8	6.90 ± 0.32	0.55 ± 0.02	32.5 ± 1.5
7.00	168	0.61 ± 0.00	0.05 ± 0.00	28.8 ± 0.0	8.45 ± 0.95	0.67 ± 0.07	39.7 ± 4.4
14.00	336	0.61 ± 0.00	0.05 ± 0.00	28.8 ± 0.0	9.33 ± 0.94	0.74 ± 0.07	43.9 ± 4.4
21.00	504	0.63 ± 0.04	0.05 ± 0.00	29.7 ± 2.1	10.25 ± 1.09	0.81 ± 0.09	48.1 ± 5.1
28.00	672	0.84 ± 0.33	0.07 ± 0.03	39.4 ± 15.4	11.37 ± 0.73	0.90 ± 0.06	53.4 ± 3.5

**Table A2:** Minimum, maximum, and mean pore sizes of the obtained composites containing antibiotics.

Name of Composite	Minimum Pore Size [mm]	Maximum Pore Size [mm]	Mean Pore Size ± Std. Dev. [mm]
PCM-MET50-CLI50	12.36	81.87	39.79 ± 12.76
PCM/CLI0250-MET25	13.66	91.02	43.45 ± 15.05
PCM/MET0250-CLI25	11.66	87.42	40.14 ± 16.17
PCM/CIP0250-MET25	16.32	106.28	52.48 ± 17.56
PCM/CIP0250-CLI25	19.32	91.22	50.74 ± 15.23
PCM/CIP0250-MET12,5-CLI12,5	21.61	81.84	45.40 ± 13.41
PCM/CIP0250-MET25-CLI25	15.18	99.83	47.13 ± 15.93
PCM/CIP0250-MET50-CLI50	19.68	104.26	53.83 ± 18.65
PCM/CIP0196/npCIP0054-MET25	16.20	83.93	47.65 ± 14.93
PCM/CIP0196/npCIP0054-CLI25	12.25	68.86	35.53 ± 11.34
PCM/CIP0125/npCIP0125-CLI25	16.36	62.26	37.29 ± 10.22
PCM/CIP0196/npMET0054-CLI25	10.58	81.00	37.24 ± 14.16

**Table A3:** Swelling ratios over time of the obtained composites.

Name of Composite	Swelling Ratio ± Std. Dev. [%]						
	Time 1 h	Time 5 h	Time 24 h	Time 168 h	Time 336 h	Time 504 h	Time 672 h
PCM-MET50-CLI50	1472.65 ± 31.58	1499.61 ± 32.03	1505.84 ± 56.09	1515.05 ± 83.81	1513.07 ± 93.07	1509.13 ± 85.75	1516.69 ± 93.31
PCM/CLI0250-MET25	1508.81 ± 65.98	1534.37 ± 50.22	1549.69 ± 55.82	1619.69 ± 55.82	1608.06 ± 39.37	1596.75 ± 47.99	1618.99 ± 84.31
PCM/MET0250-CLI25	1479.12 ± 140.04	1487.06 ± 158.28	1494.12 ± 158.25	1565.04 ± 111.19	1546.46 ± 70.62	1546.28 ± 84.73	1569.67 ± 82.45
PCM/CIP0250-MET25	1489.55 ± 121.93	1518.49 ± 79.32	1578.91 ± 56.11	1624.05 ± 87.40	1625.45 ± 105.28	1624.47 ± 96.01	1634.85 ± 140.55
PCM/CIP0250-CLI25	1262.81 ± 96.52	1265.36 ± 81.12	1363.94 ± 65.59	1509.79 ± 87.72	1498.28 ± 43.25	1506.31 ± 73.07	1508.62 ± 82.29
PCM/CIP0250-MET12,5-CLI12,5	1172.24 ± 85.04	1180.28 ± 82.53	1319.44 ± 106.98	1489.44 ± 83.22	1493.54 ± 101.67	1493.82 ± 104.02	1477.15 ± 111.17
PCM/CIP0250-MET25-CLI25	1191.72 ± 106.39	1229.02 ± 77.33	1312.88 ± 119.77	1355.08 ± 95.31	1325,49 ± 90.41	1384.81 ± 67.11	1336.85 ± 50.99
PCM/CIP0250-MET50-CLI50	1249.59 ± 112.78	1306.81 ± 70.11	1400.90 ± 122.98	1475.18 ± 81.83	1473.88 ± 59.19	1455.96 ± 94.91	1483.30 ± 97.94
PCM/CIP0196/npCIP0054-MET25	1061.22 ± 56.42	1065.77 ± 81.15	1175.34 ± 82.15	1174.25 ± 65.13	1214.87 ± 42.89	1137.35 ± 138.09	1202.57 ± 109.27
PCM/CIP0196/npCIP0054-CLI25	995.50 ± 52.60	1032.33 ± 77.90	1093.16 ± 60.94	1123.66 ± 70.62	1113.66 ± 67.54	1117.14 ± 66.42	1113.66 ± 67.54
PCM/CIP0125/npCIP0125-CLI25	917.31 ± 52.85	947.33 ± 30.25	973.99 ± 42.68	945.24 ± 36.61	989.95 ± 56.59	956.95 ± 33.06	968.70 ± 54.24
PCM/CIP0196/npMET0054-CLI25	1110.66 ± 162.12	1118.27 ± 173.95	1187.05 ± 148.47	1163.22 ± 108.61	1210.03 ± 96.38	1190.72 ± 148.17	1224.54 ± 48.93
